# Supplementary material for: New Flexible Analogues of 8-Aza-7-deazapurine Nucleosides as Potential Antibacterial Agents
Source: Int J Mol Sci. 2023 Oct 21;24(20):15421. doi: 10.3390/ijms242015421 (PMC10607158; doi:10.3390/ijms242015421)
Supplement: Supplementary file 1 [file ijms-24-15421-s001.zip › ijms-2657509-supplementary.pdf]

Supplementary Material

New flexible analogues of 8-aza-7-deazapurine nucleosides as potential antibacterial agents

Anastasia Khandazhinskaya[1Ɨ], Barbara Eletskaya[2Ɨ], Anton Mironov[2,3], Irina Konstantinova[2], Olga Efremenkova[4], Sofya Andreevskaya[5], Tatiana Smirnova[5], Larisa Chernousova[5], Evgenia Kondrashova[1], Alexander Chizhov[6], Katherine Seley-Radtke[7], Sergey Kochetkov[1], Elena Matyugina[1*]

[1]Engelhardt Institute of Molecular Biology of the Russian Academy of Sciences, 32 Vavilov St., Moscow 119991, Russia

[2]Shemyakin-Ovchinnikov Institute of Bioorganic Chemistry, Russian Academy of Sciences, Moscow 117997, Russia

[3] Peoples' Friendship University of Russia (RUDN University), Moscow, 117198, Russian Federation

[4]Gause Institute of New Antibiotics, Bol'shaya Pirogovskaya St. 11, Moscow, 119021, Russia

[5]Central Tuberculosis Research Institute, 2 Yauzskaya Alley, Moscow, 107564, Russia;

[6] Zelinsky Institute of Organic Chemistry RAS, Leninsky pr. 47, Moscow 119991, Russia;

[7]Department of Chemistry & Biochemistry, University of Maryland, Baltimore County, Baltimore, MD 21250, USA

Table of Content

HPLC and mass spectrometry data
^{1}H and ^{13}C NMR spectra of compounds **1a**, **1b**
^{1}H,^{13}C NMR and ^{15}N HMBS spectra of compounds **2-5**
^{1}H and ^{13}C NMR spectra of compounds **6-9**
Mass spectrometry data of compounds **1a**, **1b, 6-9**

HPLC and mass spectrometry data

IBCH RAS

Project Name Defaults

Reported by User: Breeze user (Breeze)

SAMPLE INFORMATION

Sample Name:	Fleximer (2)	Acquired By:	Breeze
Sample Type:	Unknown	Date Acquired:	20.03.2023 18:54:10 GMT-4
Vial:	1	Acq. Method:	100B_dual_230nm_05ml
Injection #:	2	Date Processed:	20.03.2023 19:15:34 GMT-4
Injection Volume:	20,00 ul	Channel Name:	W2489 ChB
Run Time:	20,00 Minutes	Channel Desc.:	W2489 ChB 254nm
Column Type:		Sample Set Name	

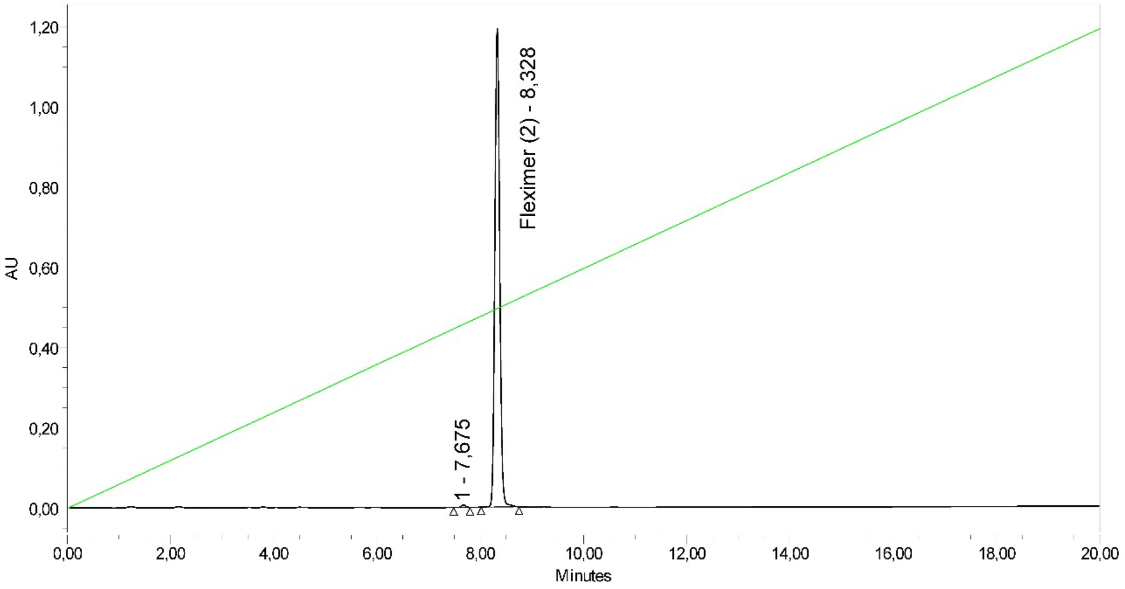

	Peak Name	RT (min)	Area (µV*sec)	% Area	Height (µV)	% Height
1	Peak 1	7,675	35388	0,46	5740	0,48
2	Fleximer (2)	8,328	7637798	99,54	1194687	99,52

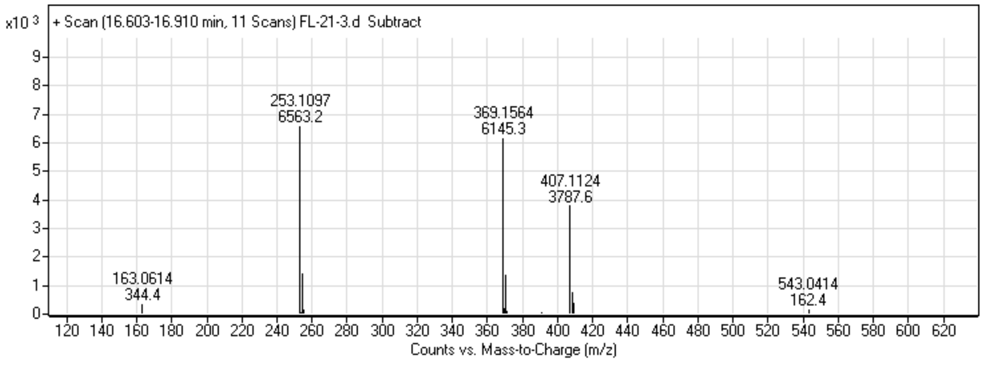

1-(β-D-2’-Deoxyribofuranosyl)-4-(4-benzyloxypyrimidin-5-yl)pyrazole (**2**)

IBCH RAS

Project Name Defaults

Reported by User: Breeze user (Breeze)

SAMPLE INFORMATION

Sample Name:	Fleximer (3)	Acquired By:	Breeze
Sample Type:	Unknown	Date Acquired:	02.03.2023 13:40:20 GMT-4
Vial:	1	Acq. Method:	100B_dual_230nm_05ml
Injection #:	1	Date Processed:	20.03.2023 18:59:13 GMT-4
Injection Volume:	15,00 ul	Channel Name:	W2489 ChB
Run Time:	20,00 Minutes	Channel Desc.:	W2489 ChB 254nm
Column Type:		Sample Set Name	

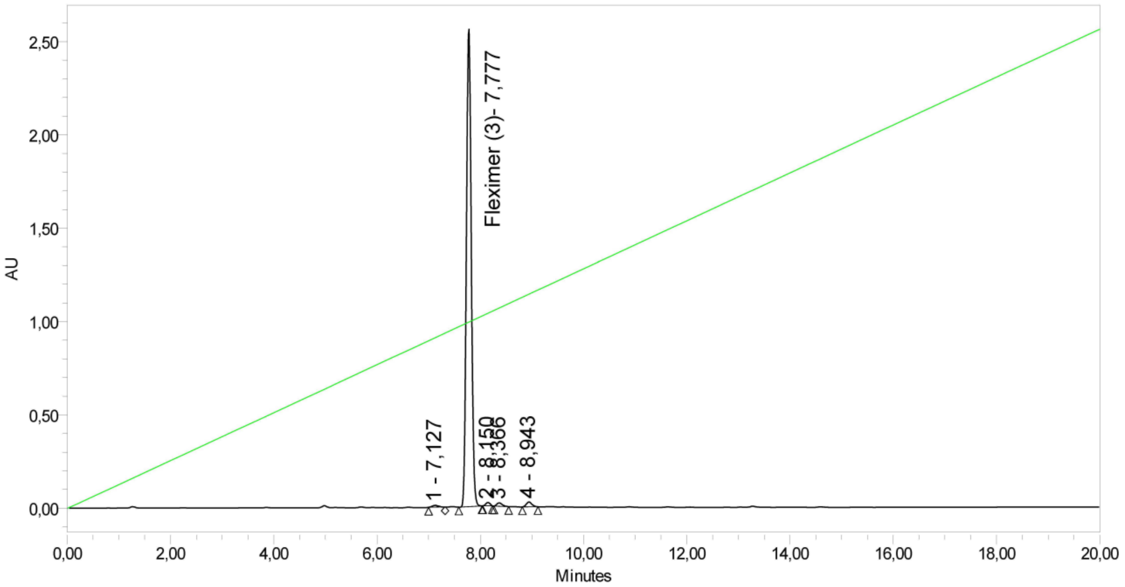

	Peak Name	RT (min)	Area (µV*sec)	% Area	Height (µV)	% Height
1	1	7,127	81342	0,48	9355	0,35
2	Fleximer (3)	7,777	16480090	97,03	2567521	97,29
3	2	8,150	100170	0,59	17952	0,68
4	3	8,366	129334	0,76	17959	0,68
5	4	8,943	194086	1,14	26299	1,00

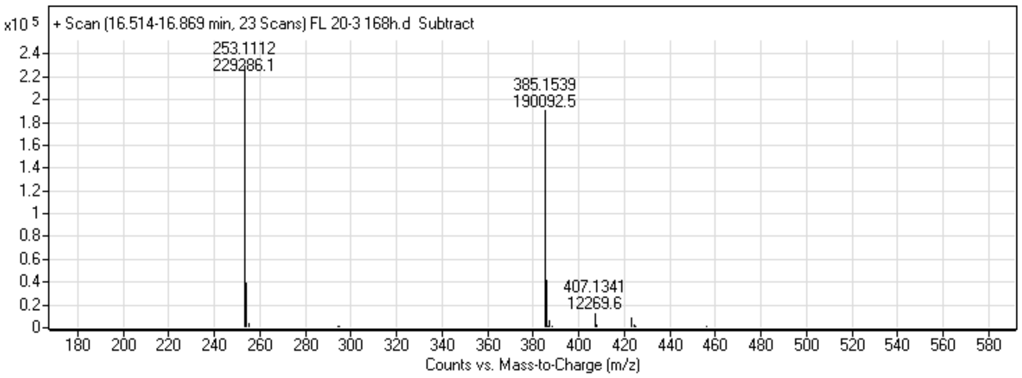

1-(β-D-Ribofuranosyl)-4-(4-benzyloxypyrimidin-5-yl)pyrazole (**3**)

IBCH RAS

Project Name Defaults

Reported by User: Breeze user (Breeze)

Breeze 2 HPLC System

SAMPLE INFORMATION

Sample Name:	Fleximer (4)	Acquired By:	Breeze
Sample Type:	Unknown	Date Acquired:	01.06.2023 16:22:01 GMT-4
Vial:	1	Acq. Method:	100B_dual_230nm_05ml
Injection #:	1	Date Processed:	01.06.2023 16:50:42 GMT-4
Injection Volume:	10,00 ul	Channel Name:	W2489 ChB
Run Time:	20,00 Minutes	Channel Desc.:	W2489 ChB 254nm
Column Type:		Sample Set Name	

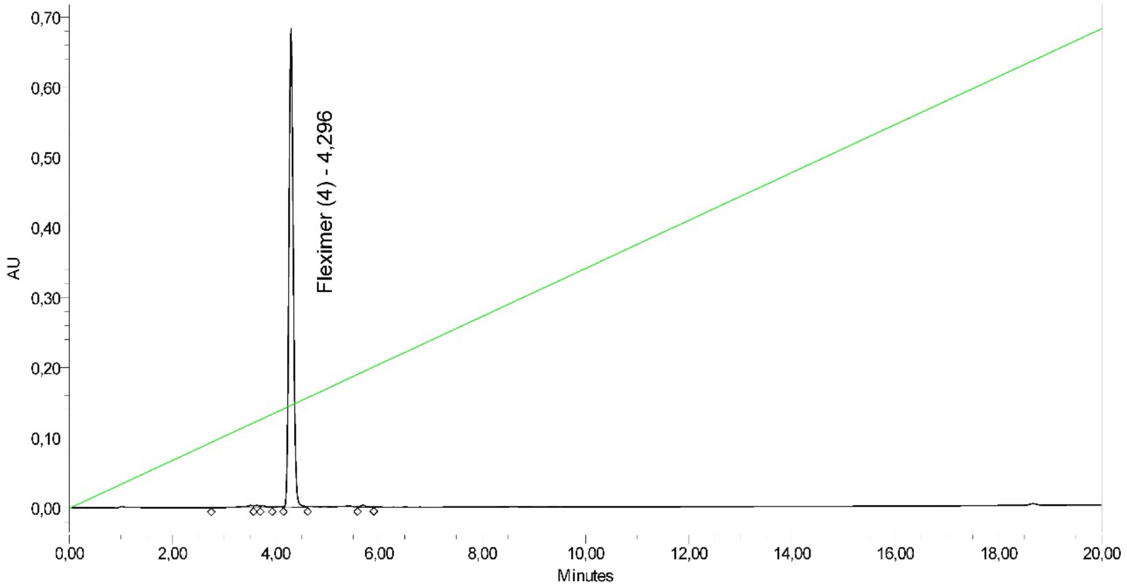

	Peak Name	RT (min)	Area (µV*sec)	% Area	Height (µV)	% Height
1	1	3,516	54322	1,31	3309	0,48
2	2	3,628	23993	0,58	3531	0,51
3	3	3,747	23156	0,56	2950	0,42
4	Fleximer (4)	4,296	4011516	96,84	682065	98,12
5	5	5,690	29295	0,71	3246	0,47

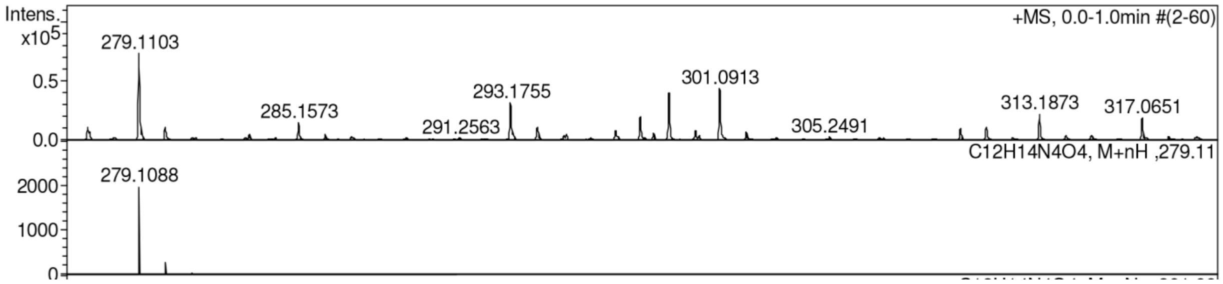

1-(β-D-2’-Deoxyribofuranosyl)-4-(pyrimidin-4(3*H*)-on-5-yl)pyrazole (**4**)

IBCH RAS

Project Name Defaults

Reported by User: Breeze user (Breeze)

SAMPLE INFORMATION

Sample Name:	Fleximer (5)	Acquired By:	Breeze
Sample Type:	Unknown	Date Acquired:	29.11.2022 17:06:42 GMT-4
Vial:	31	Acq. Method:	50B_dual_280_05ml
Injection #:	1	Date Processed:	14.06.2023 13:29:44 GMT-4
Injection Volume:	10,00 ul	Channel Name:	W2489 ChB
Run Time:	18,00 Minutes	Channel Desc.:	W2489 ChB 280nm
Column Type:		Sample Set Name	Fleximers

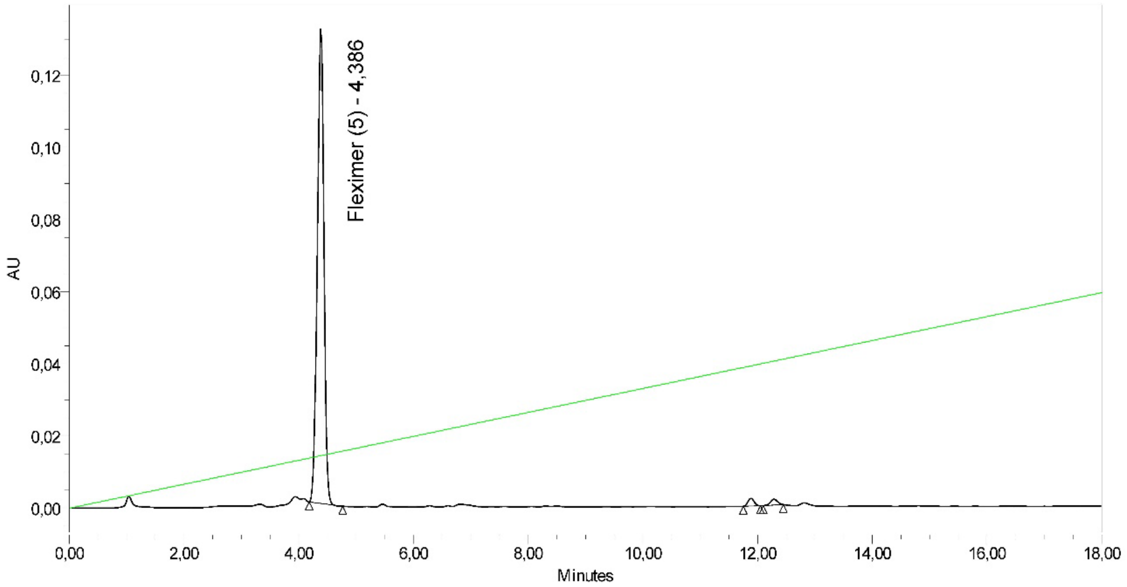

	Peak Name	RT (min)	Area (µV*sec)	% Area	Height (µV)	% Height
1	Fleximer (5)	4,386	1049920	97,16	131302	97,18
2	3	11,884	16271	1,51	2141	1,59
3	4	12,286	14453	1,34	1664	1,23

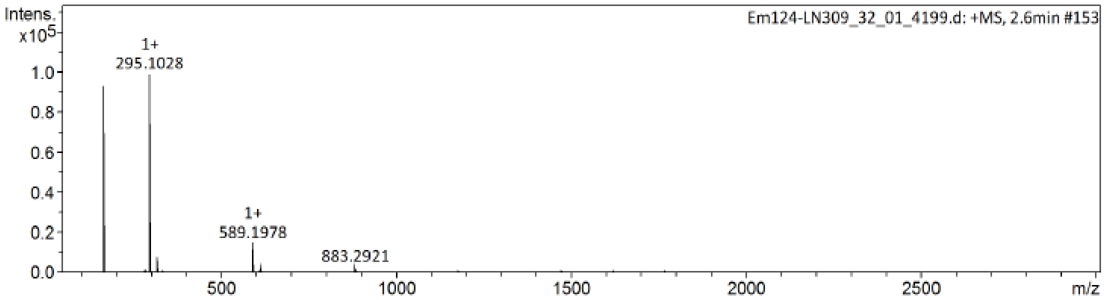

1-(β-D-Ribofuranosyl)-4-(pyrimidin-4(3*H*)-on-5-yl)pyrazole (**5**)

HPLC of side products from transglycosylation reaction mixture (fleximer riboside synthesis)

IBCH RAS

Project Name Defaults

Reported by User: Breeze user (Breeze)

SAMPLE INFORMATION

Sample Name:	FL-11/1 1h	Acquired By:	Breeze
Sample Type:	Unknown	Date Acquired:	24.05.2021 15:58:16 GMT-4
Vial:	7	Acq. Method:	Fleximers_0_15B_10 min
Injection #:	1	Date Processed:	02.08.2023 13:42:16 GMT-4
Injection Volume:	10,00 ul	Channel Name:	W2489 ChB
Run Time:	15,00 Minutes	Channel Desc.:	W2489 ChB 280nm
Column Type:		Sample Set Name	Fleximers

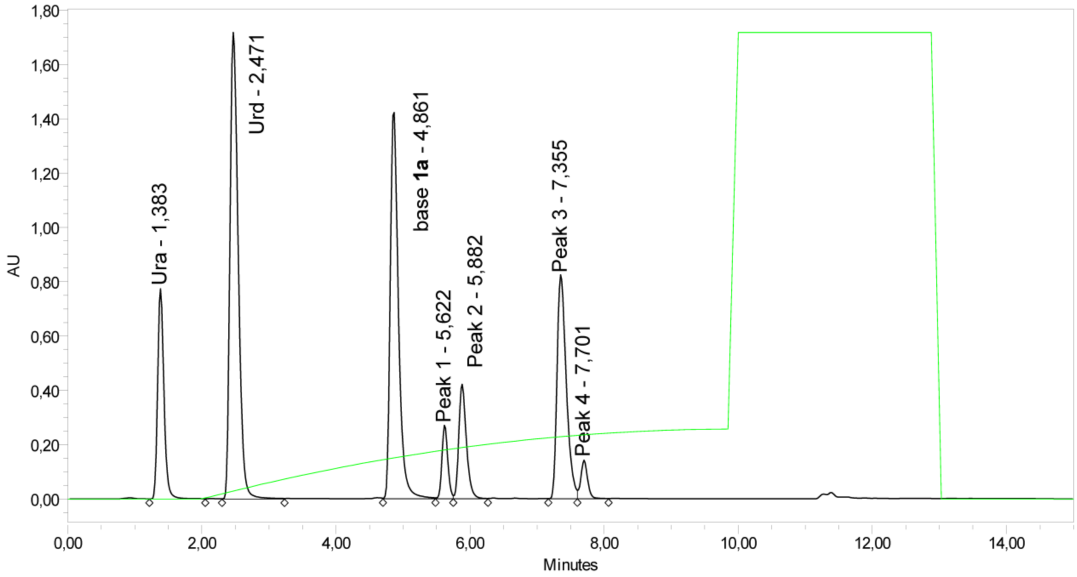

	Peak Name	RT (min)	Area (µV*sec)	% Area	Height (µV)	% Height
1	Ura	1,383	5043469	11,01	769387	13,80
2	Urd	2,471	14716294	32,14	1720877	30,86
3	base **1a**	4,861	12149922	26,53	1426226	25,58
4	Peak 1	5,622	1632296	3,56	270525	4,85
5	Peak 2	5,882	3334549	7,28	420576	7,54
6	Peak 3	7,355	7906597	17,27	826918	14,83
7	Peak 4	7,701	1008487	2,20	141244	2,53

Report Method: Gradient Overlay Report ASC

Printed: 02.08.2023 13:45:09 Etc/GMT-4

HPLC of side products from transglycosylation reaction mixture (fleximer 2'-deoxyriboside synthesis)

IBCH RAS

Project Name Defaults

Reported by User: Breeze user (Breeze)

Breeze 2 HPLC System

SAMPLE INFORMATION

Sample Name:	FL-13/1 1h	Acquired By:	Breeze
Sample Type:	Unknown	Date Acquired:	24.05.2021 16:29:22 GMT-4
Vial:	7	Acq. Method:	Fleximers_0_15B_10 min
Injection #:	1	Date Processed:	02.08.2023 13:51:27 GMT-4
Injection Volume:	10,00 ul	Channel Name:	W2489 ChB
Run Time:	15,00 Minutes	Channel Desc.:	W2489 ChB 280nm
Column Type:		Sample Set Name	Fleximers

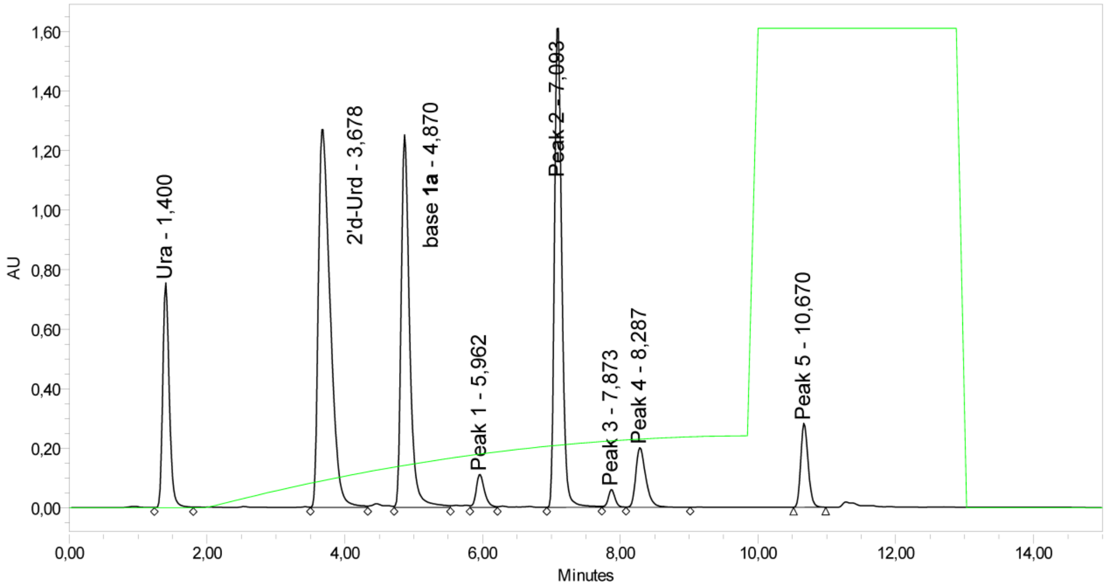

	Peak Name	RT (min)	Area (µV*sec)	% Area	Height (µV)	% Height
1	Ura	1,400	4974523	10,30	751107	13,54
2	2'd-Urd	3,678	15205213	31,47	1272912	22,94
3	base 1a	4,870	10682803	22,11	1251423	22,55
4	Peak 1	5,962	938773	1,94	110914	2,00
5	Peak 2	7,093	11945274	24,72	1619756	29,19
6	Peak 3	7,873	417468	0,86	60052	1,08
7	Peak 4	8,287	2045992	4,23	200852	3,62
8	Peak 5	10,670	2102629	4,35	282025	5,08

Report Method: Gradient Overlay Report ASC

Printed: 02.08.2023 13:52:28 Etc/GMT-4

NMR spectra of compounds

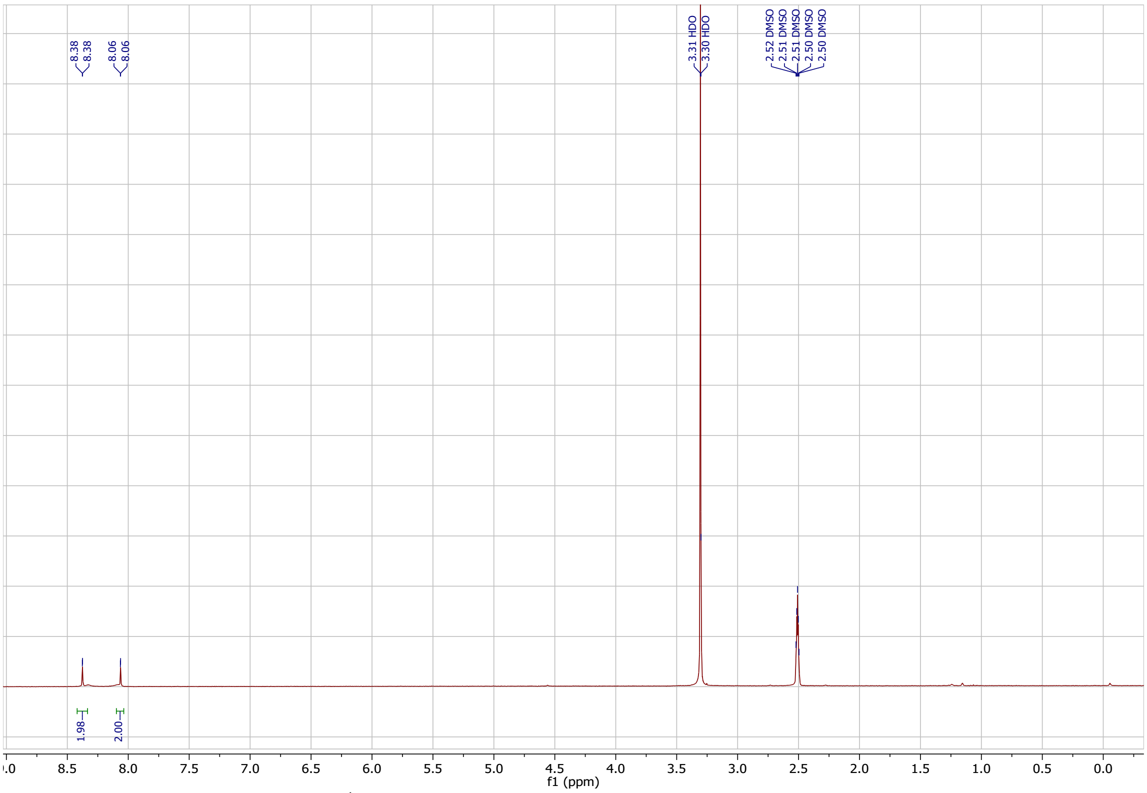

^{1}H NMR spectrum of **1a** in CD_3OD

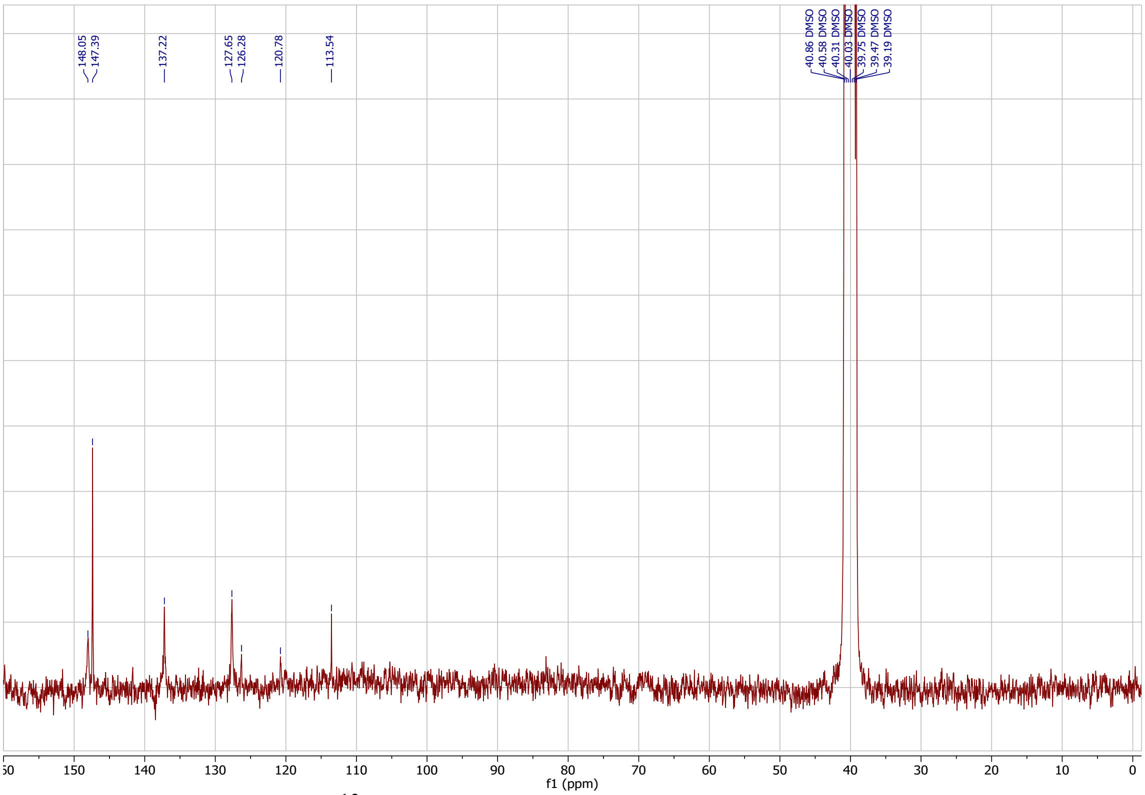

^{13}C NMR spectrum of **1a** in CD_3OD

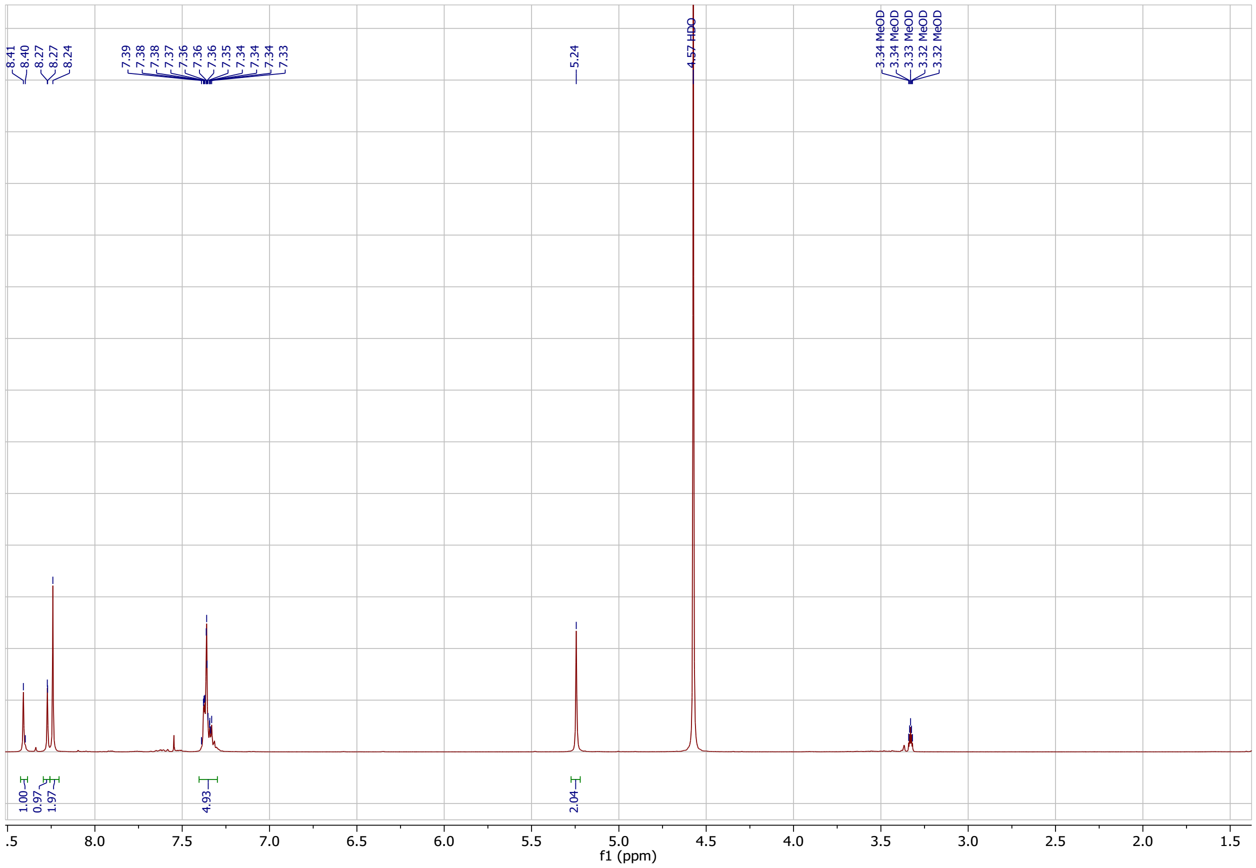

^{1}H NMR spectrum of **1b** in CD_3OD

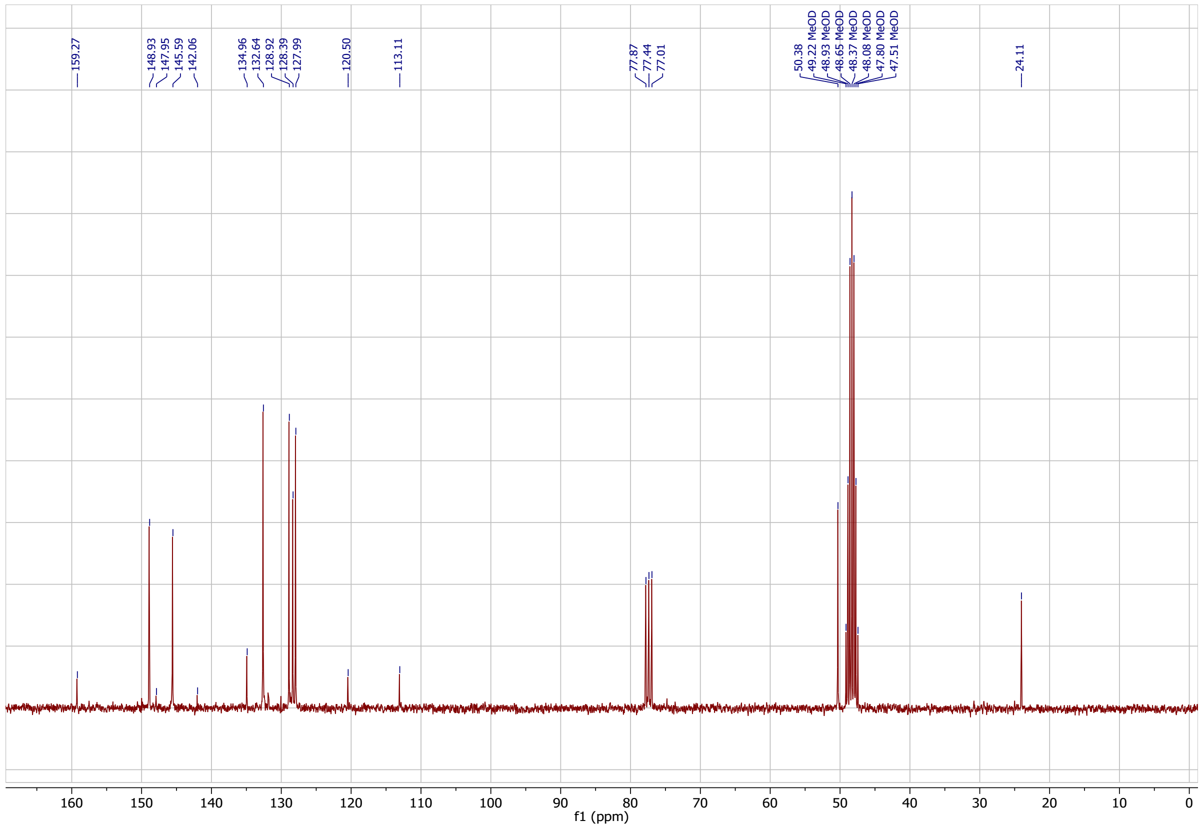

^{13}C NMR spectrum of **1b** in CD_3OD

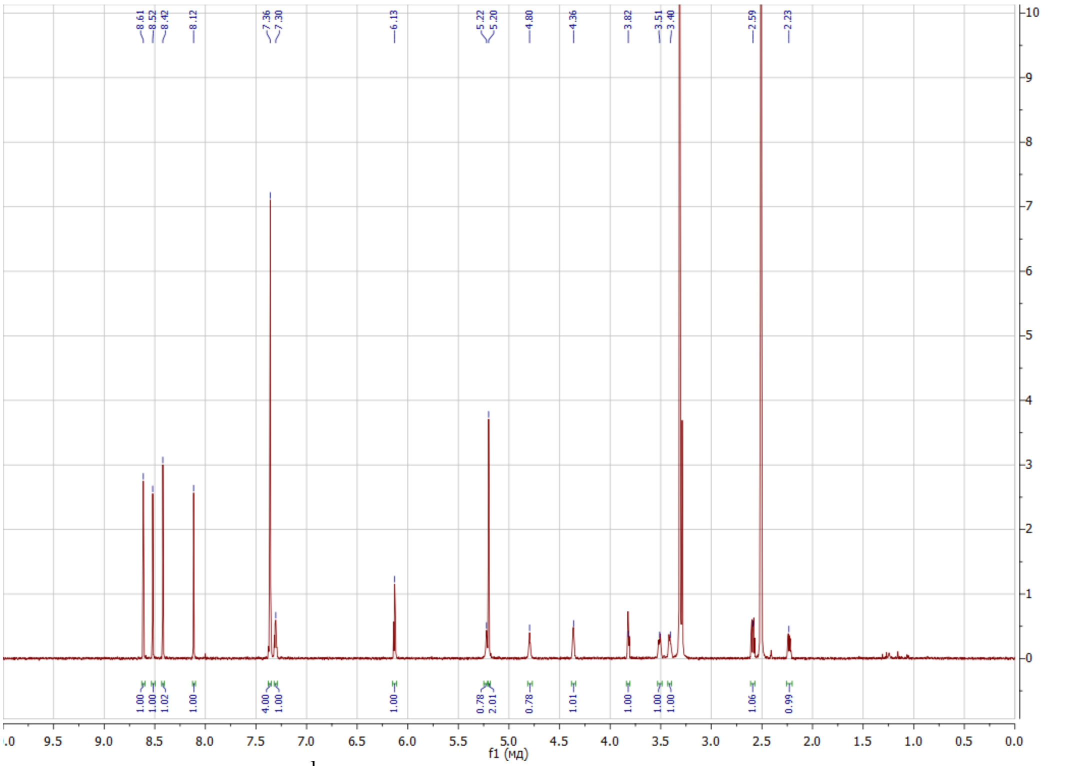

^{1}H NMR spectrum of **2** in DMSO-*d*6

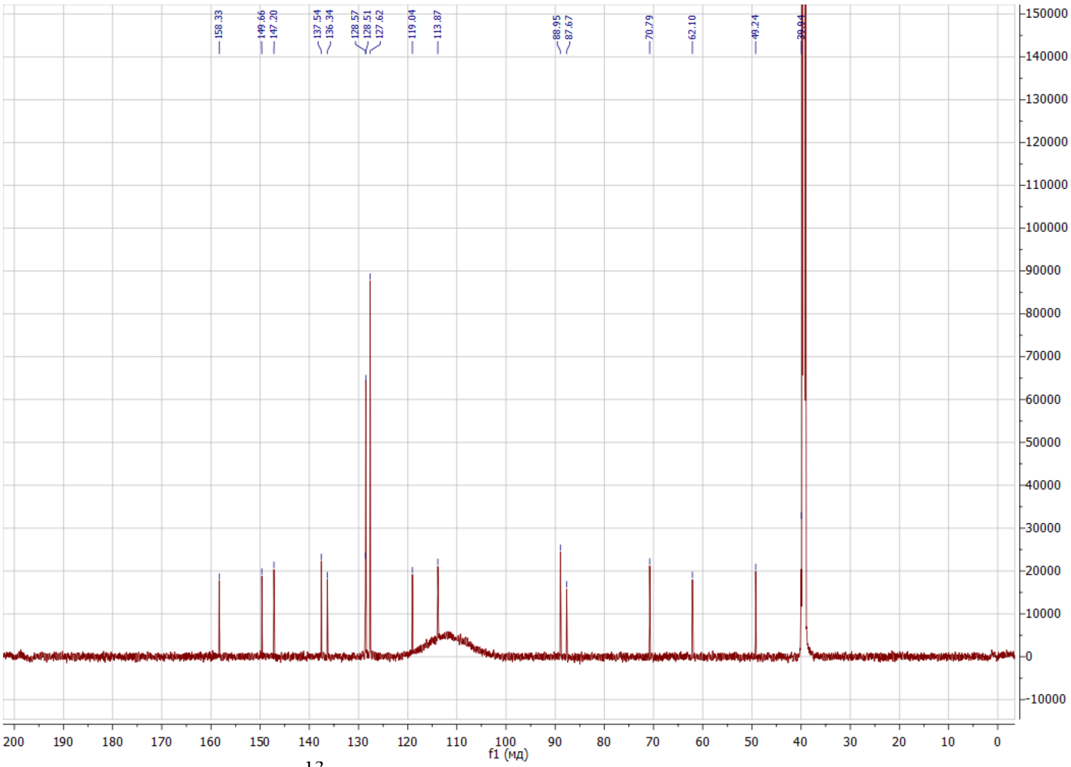

^{13}C NMR spectrum of **2** in DMSO-*d*6

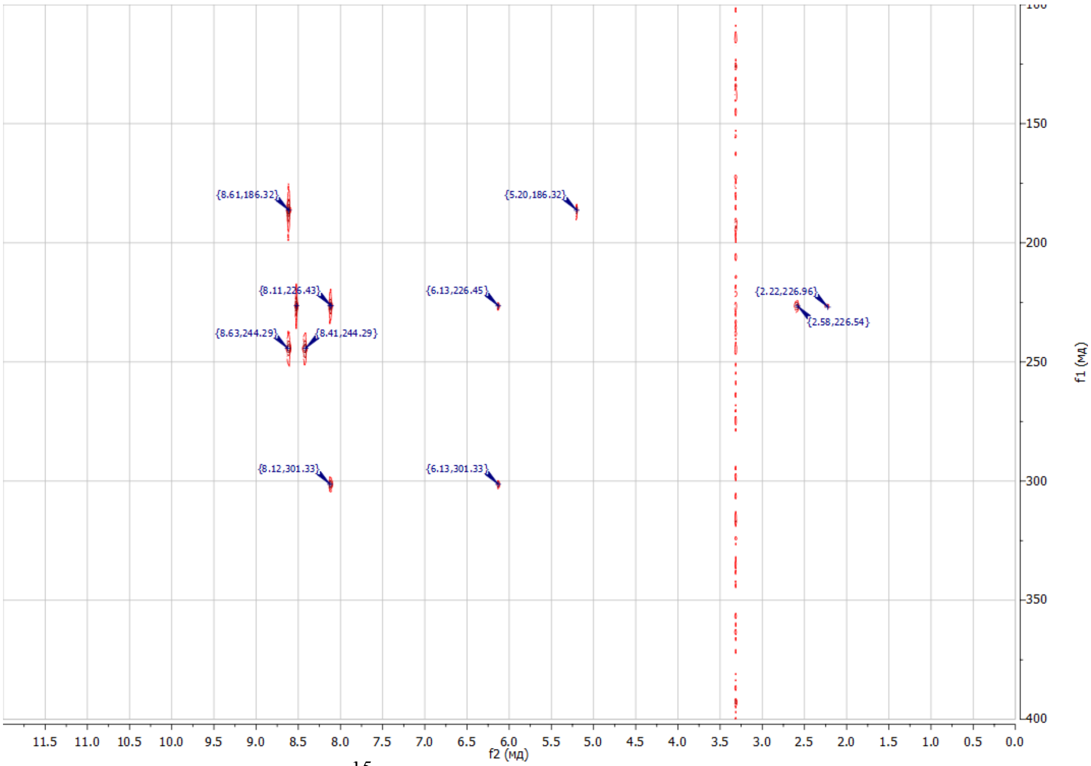

HMBS ^{15}N NMR spectrum of **2** in DMSO-*d*6

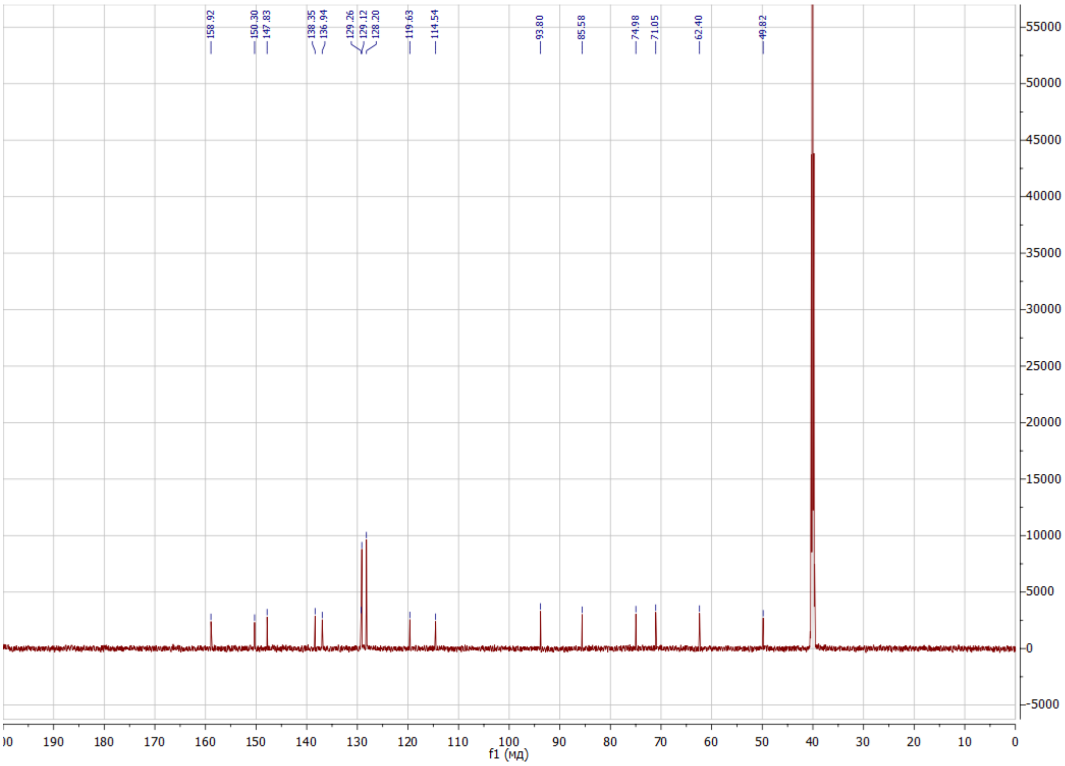

^{1}H NMR spectrum of **3** in DMSO-*d*6

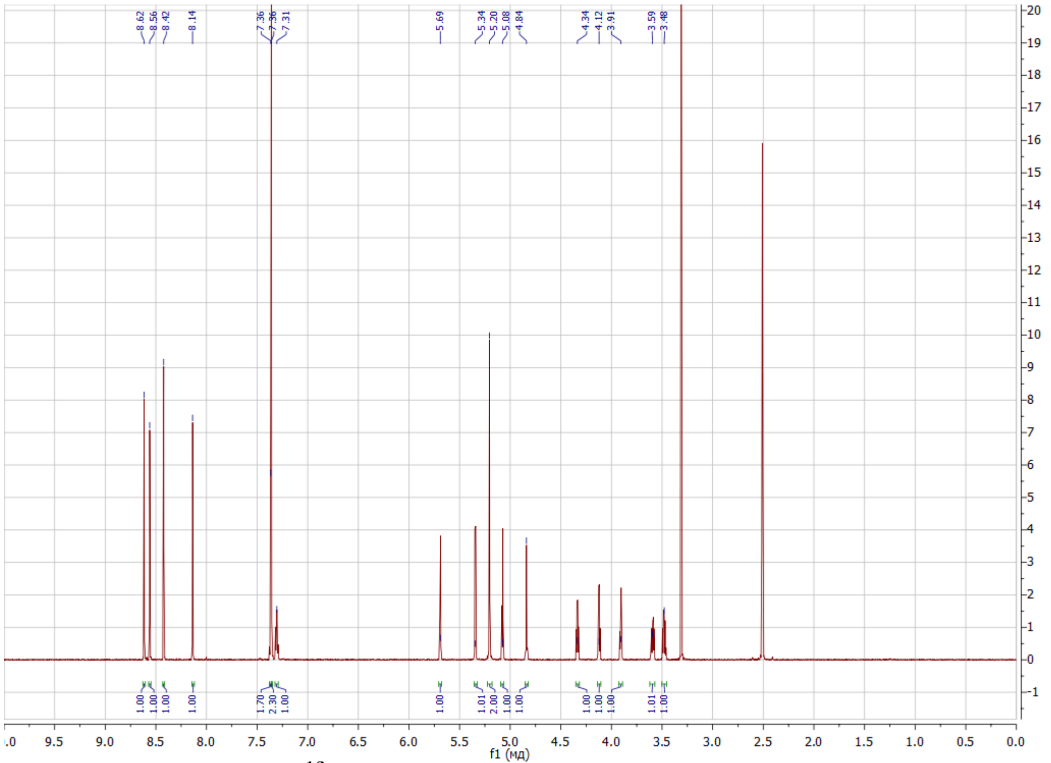

^{13}C NMR spectrum of **3** in DMSO-*d6*

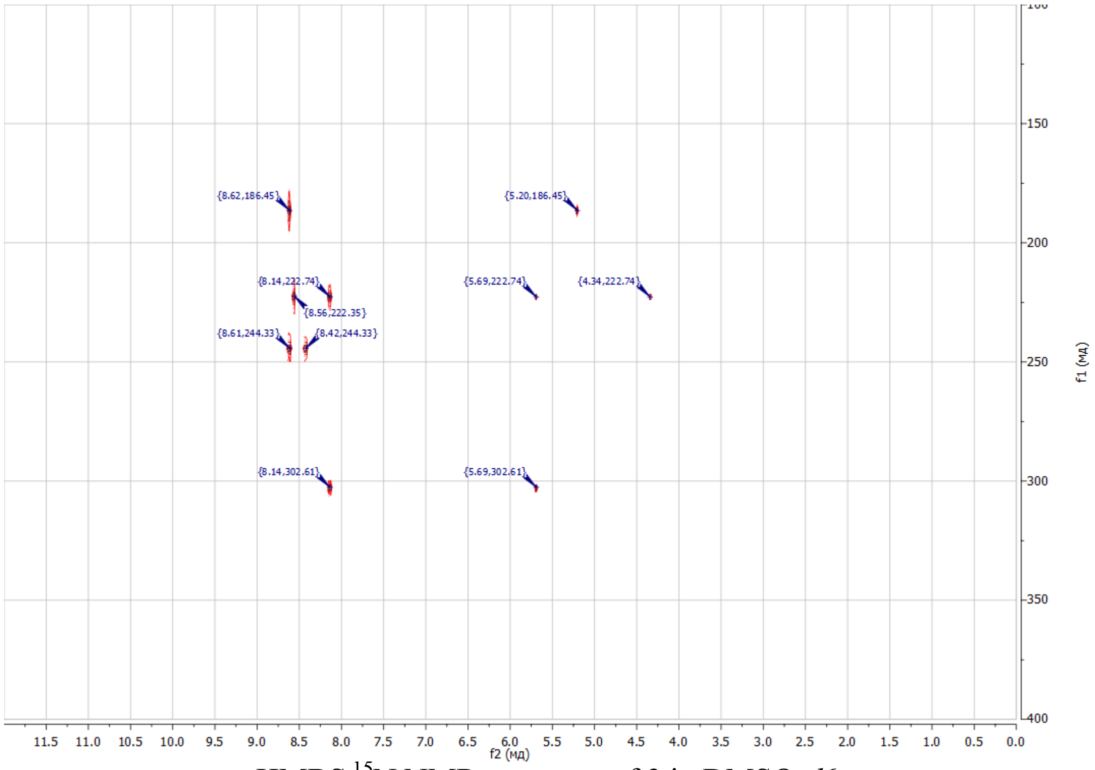

HMBS ^{15}N NMR spectrum of **3** in DMSO-*d6*

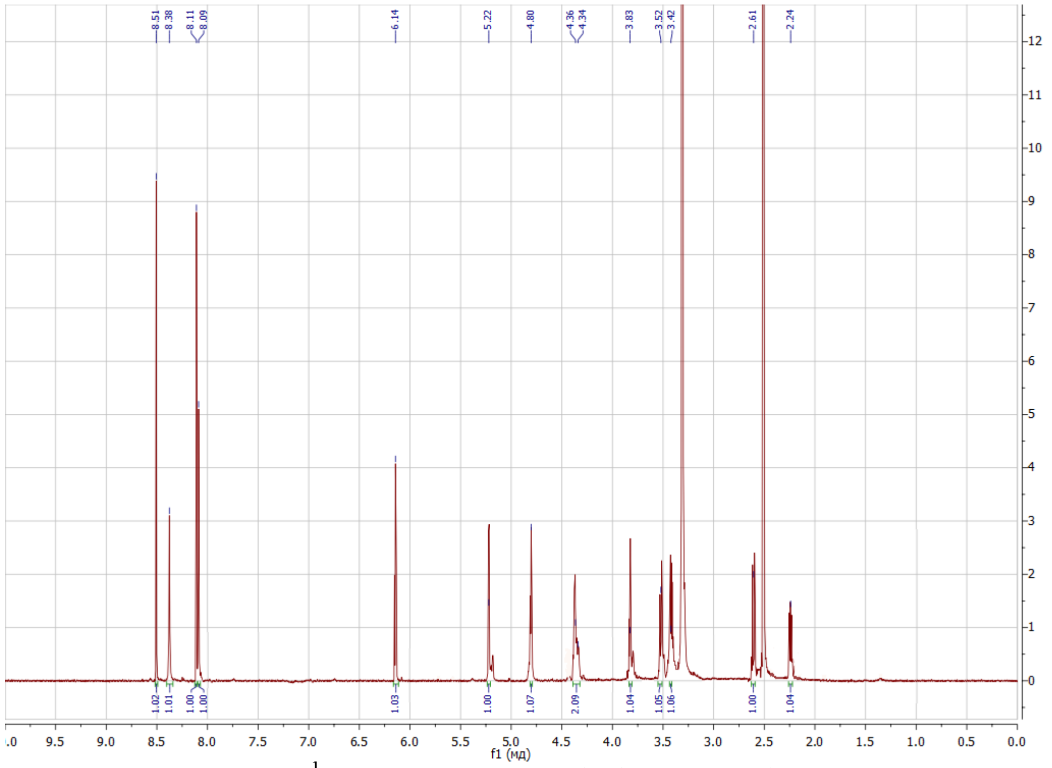

^{1}H NMR spectrum of **4** in DMSO-*d*6

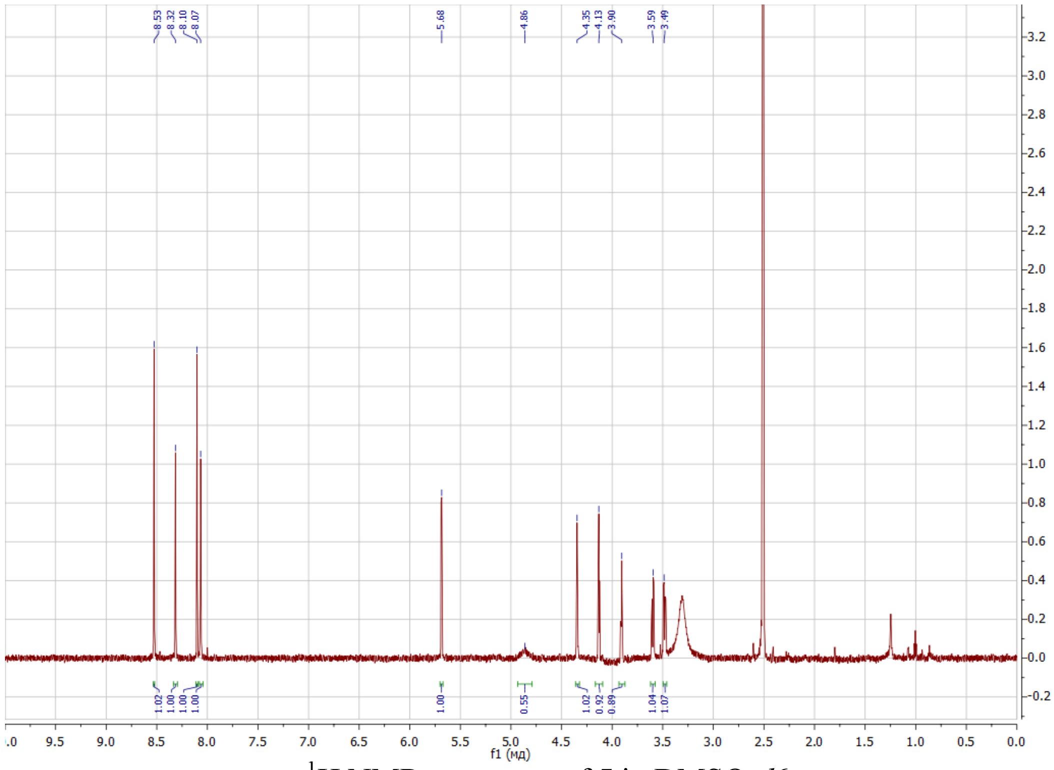

^{1}H NMR spectrum of **5** in DMSO-*d*6

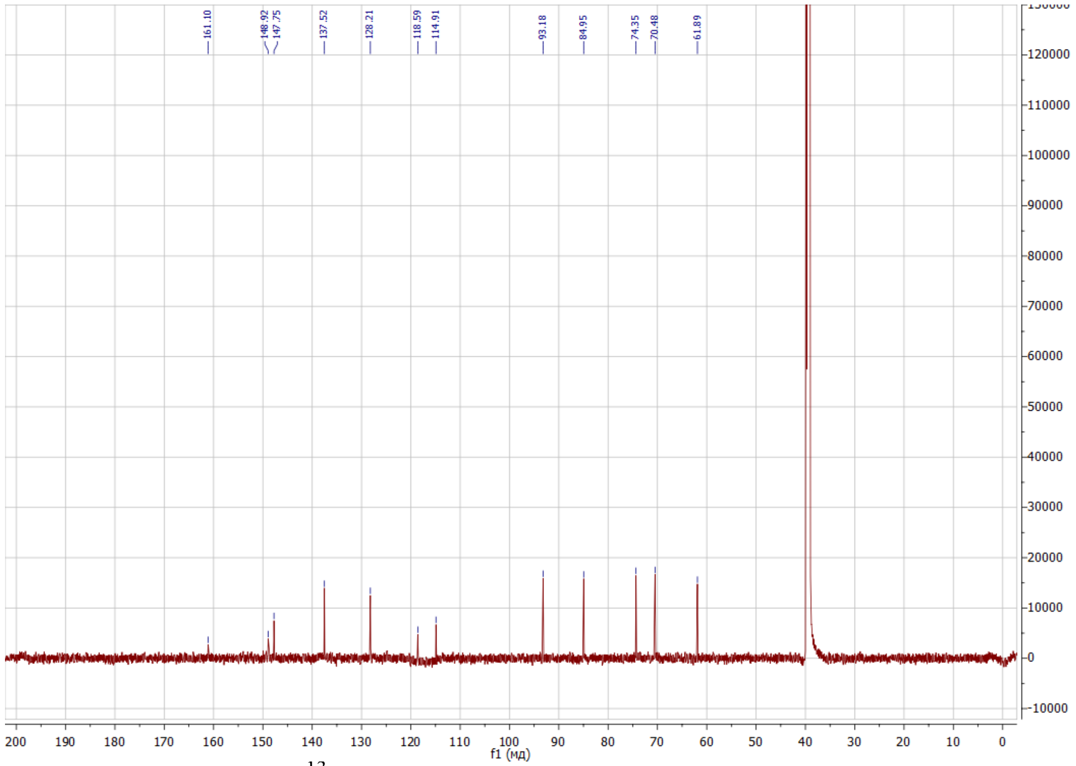

^{13}C NMR spectrum of **5** in DMSO-*d6*

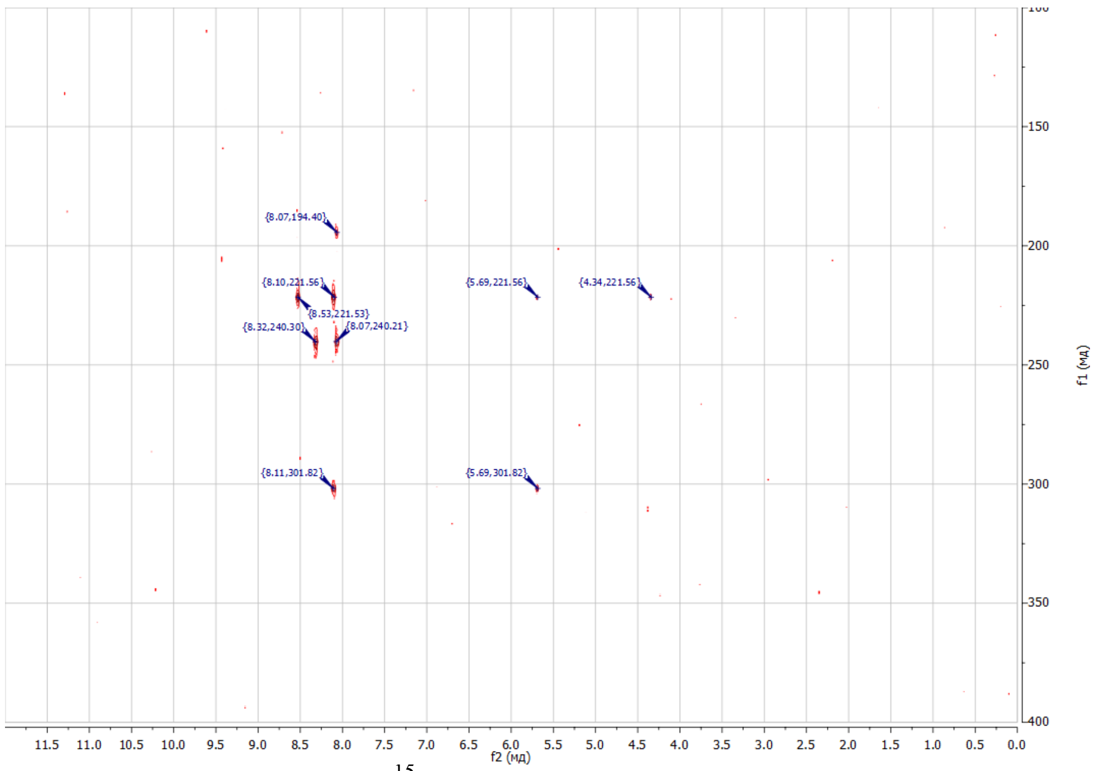

HMBS ^{15}N spectrum of **5** in DMSO-*d6*

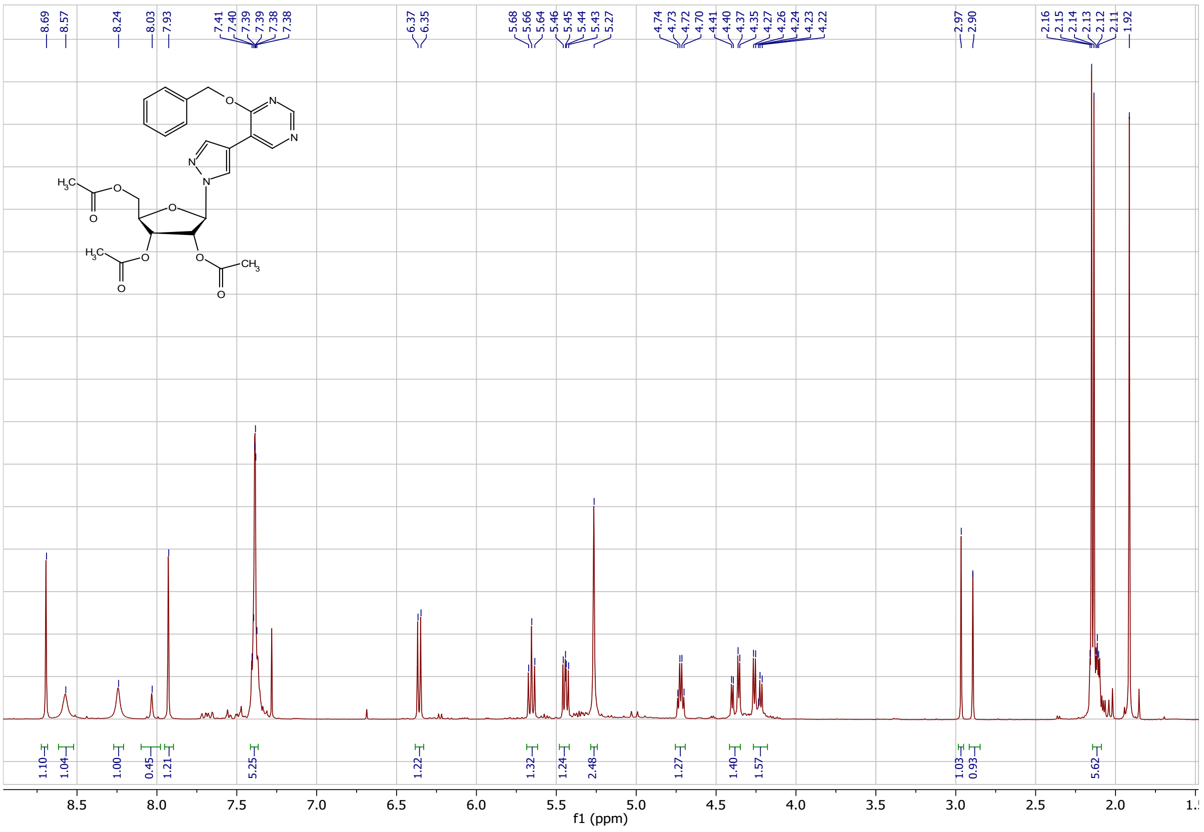

^{1}H NMR spectrum of 1-(β-D-(2’,3’,5’-triacetylribofuranosyl))-4-(4-benzyloxypyrimidin-5-yl)pyrazole in $CDCl_3$

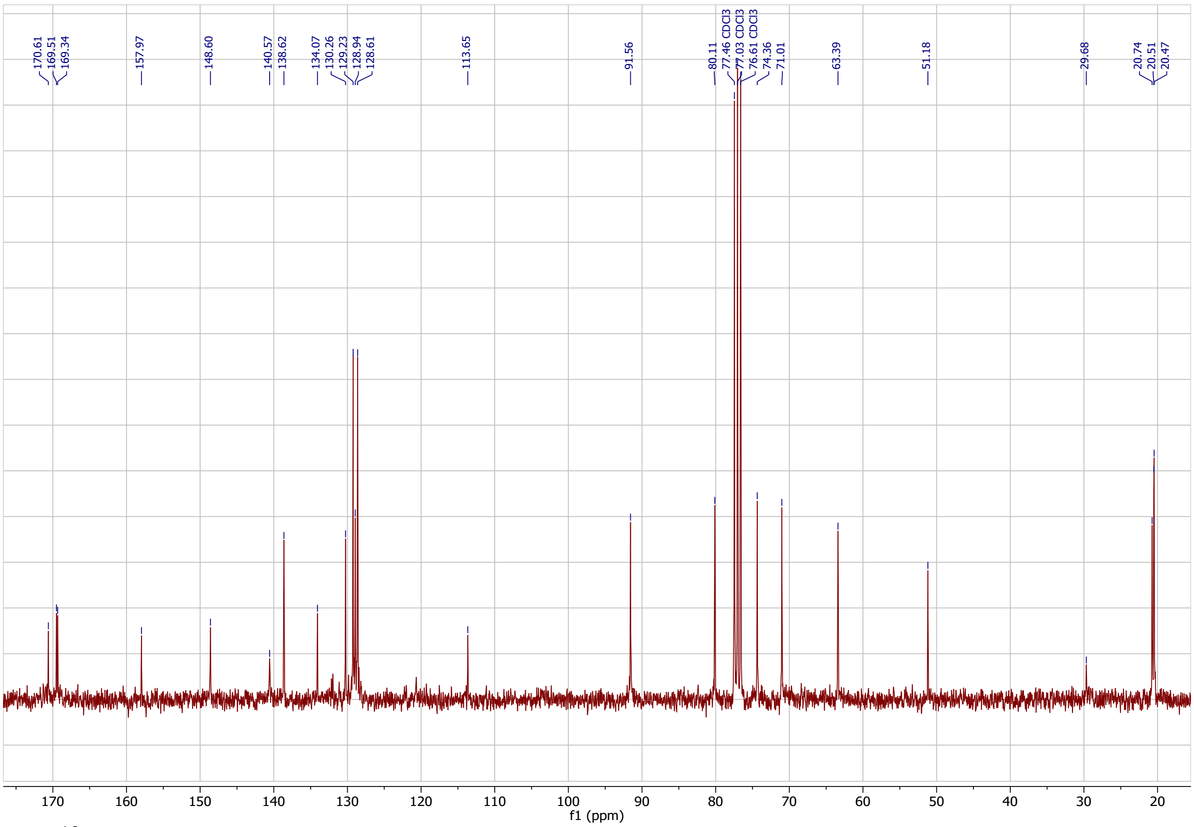

^{13}C NMR spectrum of 1-(β-D-(2’,3’,5’-triacetylribofuranosyl))-4-(4-benzyloxypyrimidin-5-yl)pyrazole in $CDCl_3$

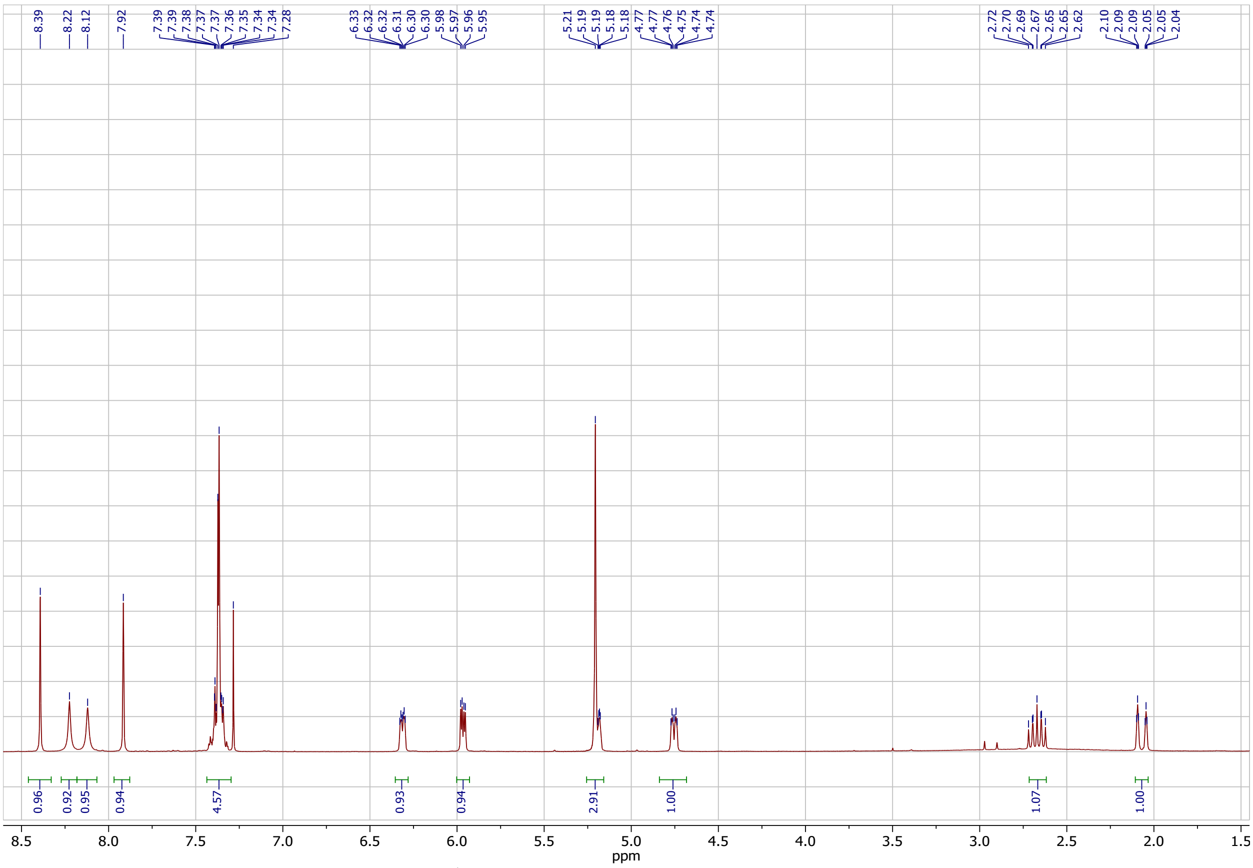

^{1}H NMR spectrum of **6** in $CDCl_3$

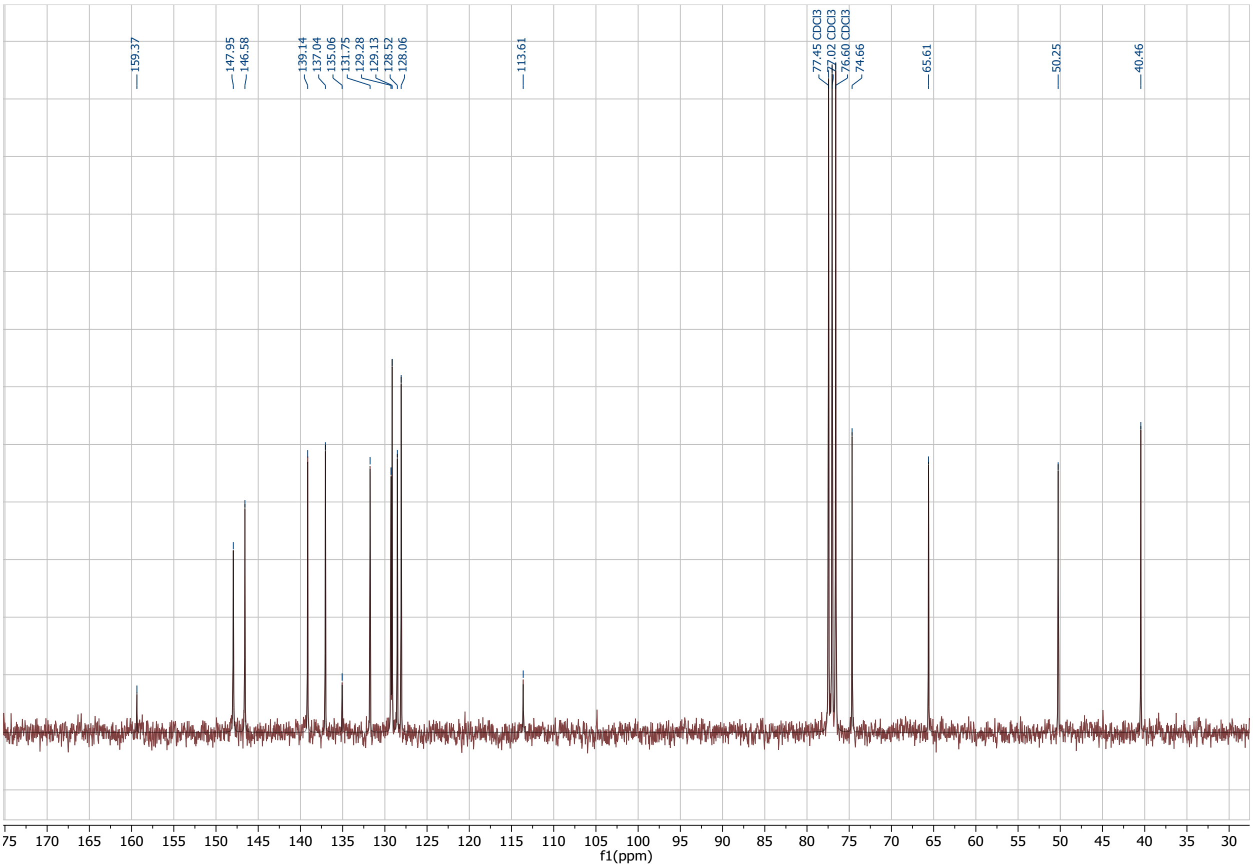

^{13}C NMR spectrum of **6** in $CDCl_3$

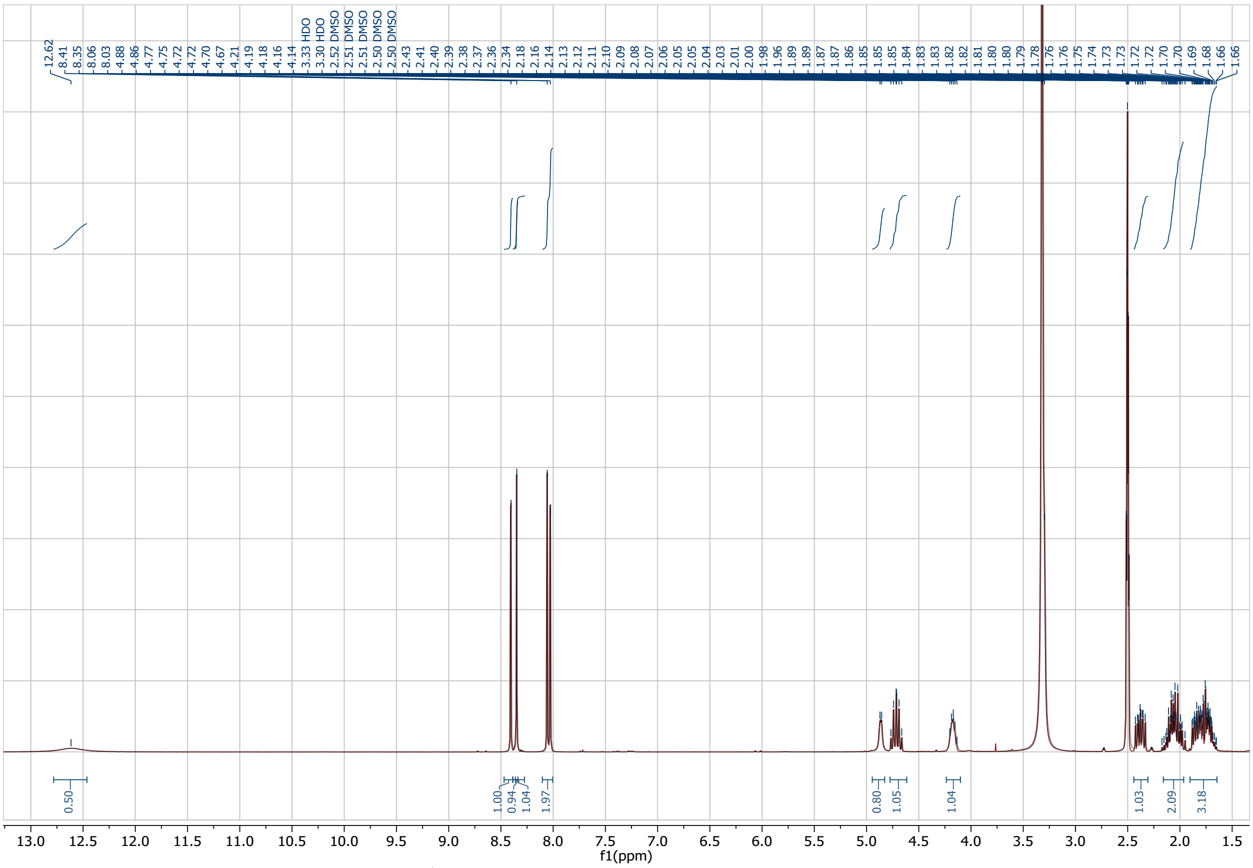

^{1}H NMR spectrum of **7** in DMSO-*d*6

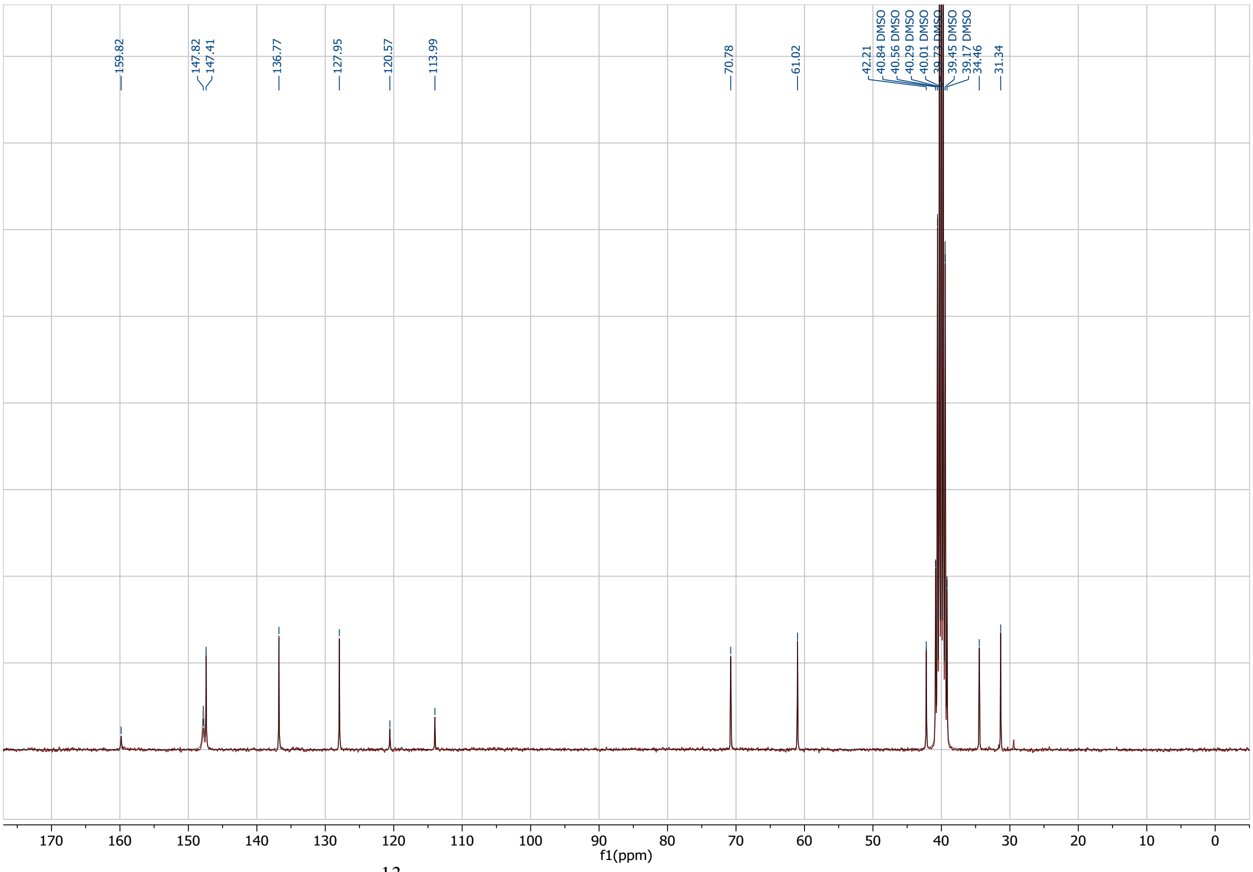

^{13}C NMR spectrum of **7** in DMSO-*d*6

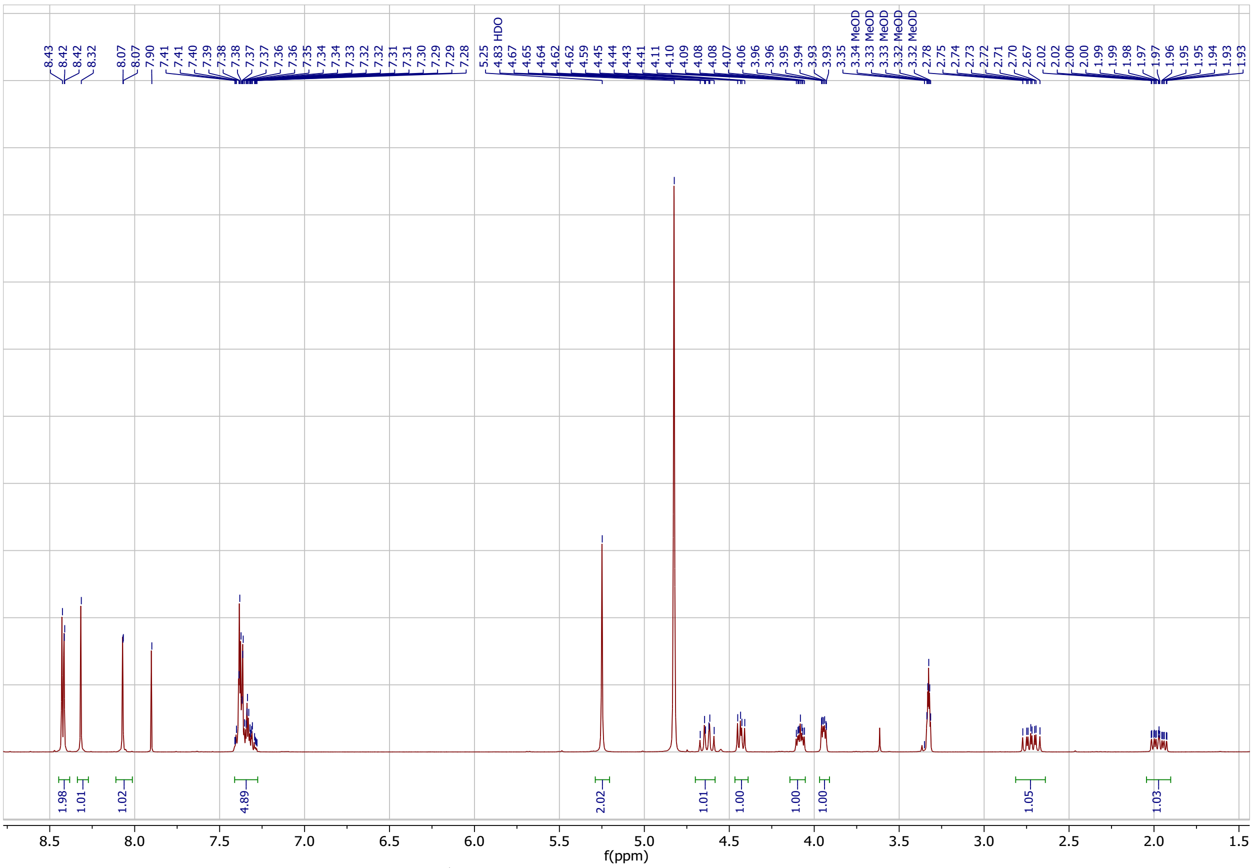

^{1}H NMR spectrum of **8** in CD_3OD

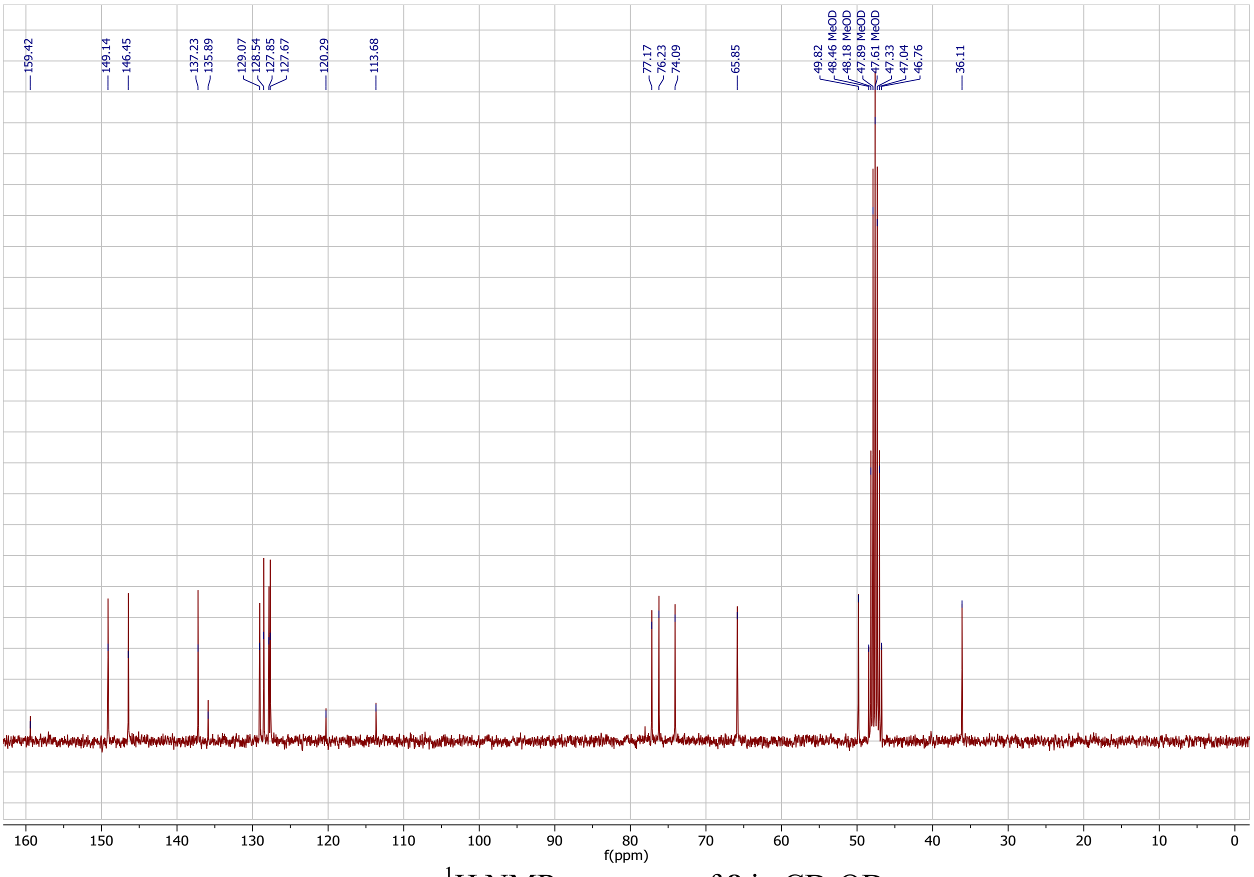

^{1}H NMR spectrum of **8** in CD_3OD

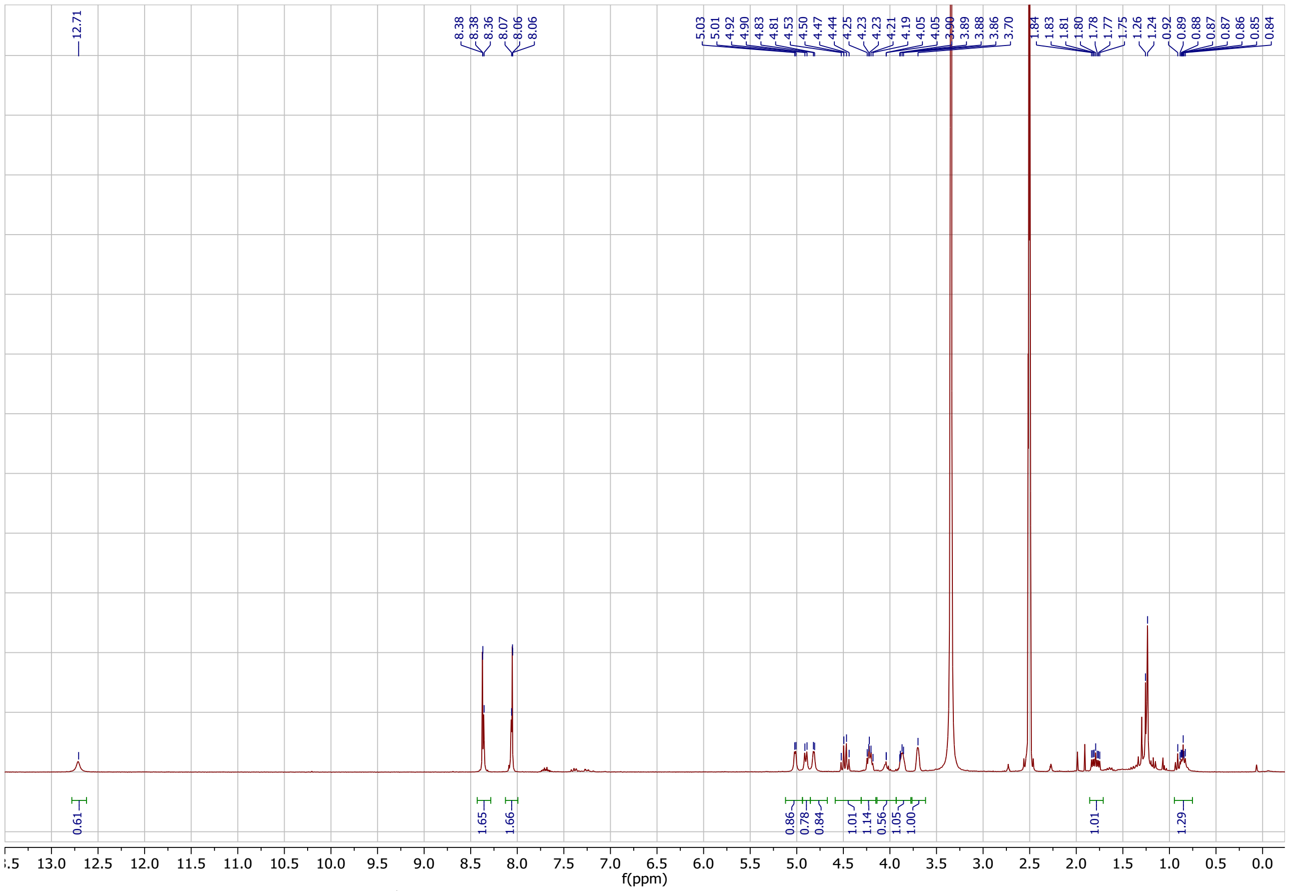

^{1}H NMR spectrum of **9** in DMSO-*d6*

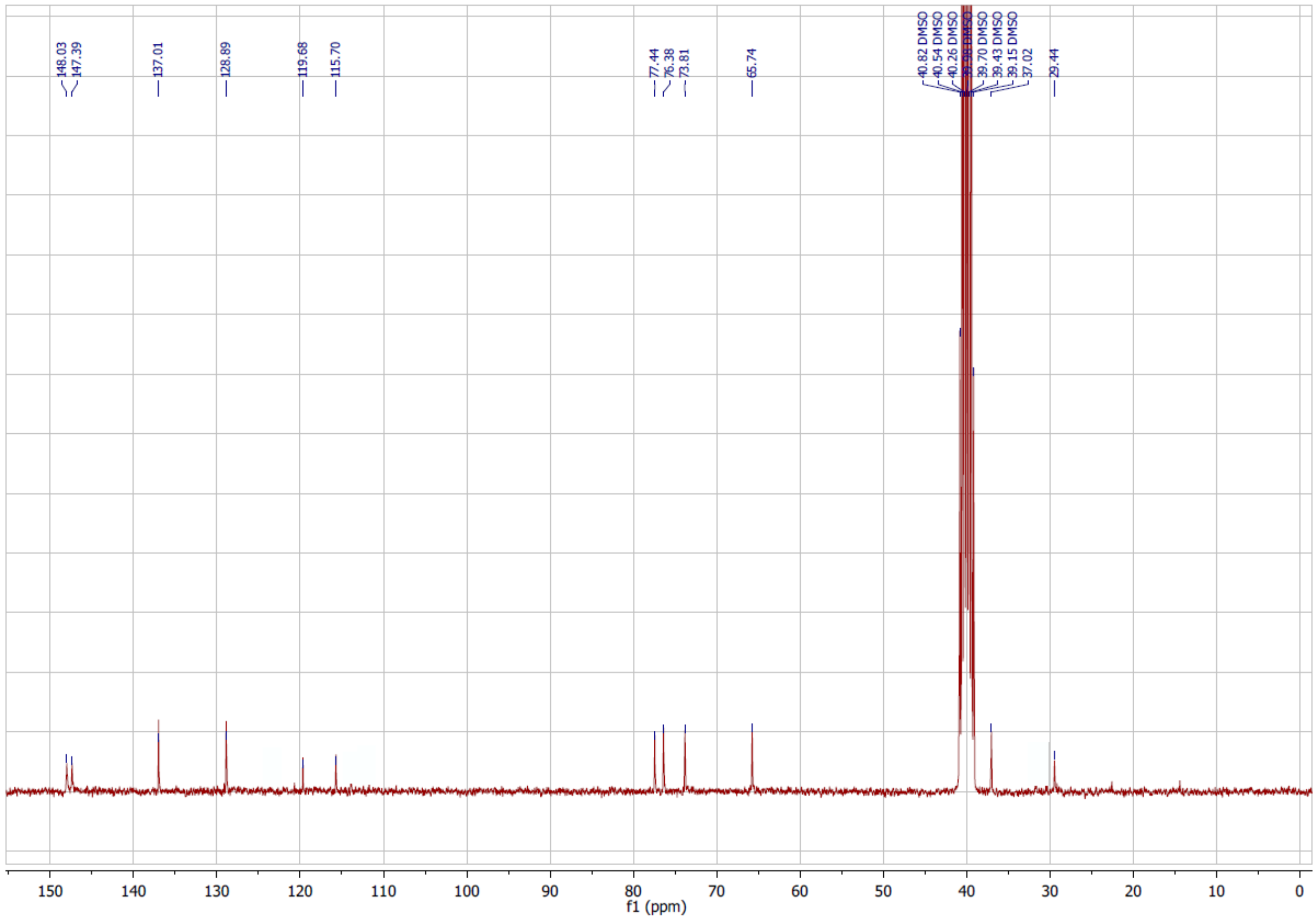

^{13}C NMR spectrum of **9** in DMSO-*d6*

HRMS spectrum of **1a**

Display Report

Analysis Info

Analysis Name	D:\Data\EM-98(LN-201)_11_01_3659.d	Acquisition Date	11/12/2020 3:05:57 PM
Method	la_2.2_small.m	Operator	BDAL@DE
Sample Name	1a	Instrument	compact 8255754.20088
Comment			

Acquisition Parameter

Source Type	ESI	Ion Polarity	Positive	Set Nebulizer	0.4 Bar
Focus	Active	Set Capillary	4500 V	Set Dry Heater	180 °C
Scan Begin	50 m/z	Set End Plate Offset	-500 V	Set Dry Gas	6.0 l/min
Scan End	3000 m/z	Set Charging Voltage	2000 V	Set Divert Valve	Source
		Set Corona	0 nA	Set APCI Heater	0 °C

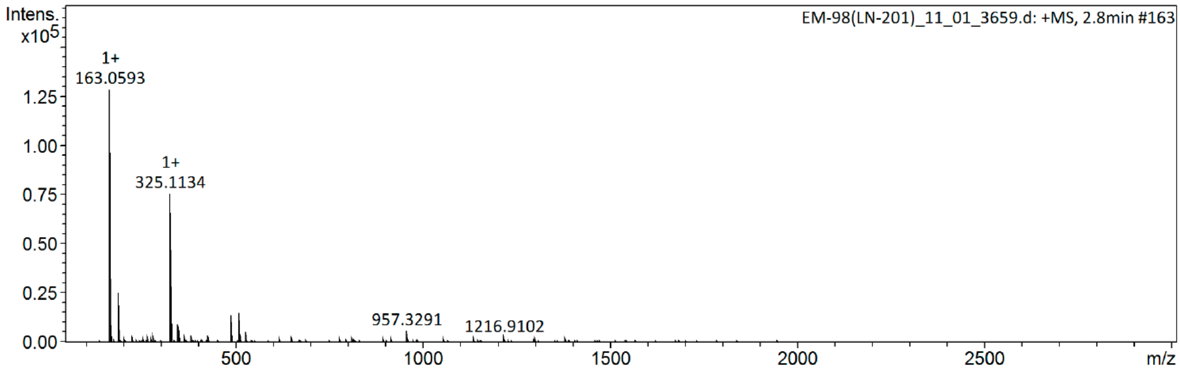

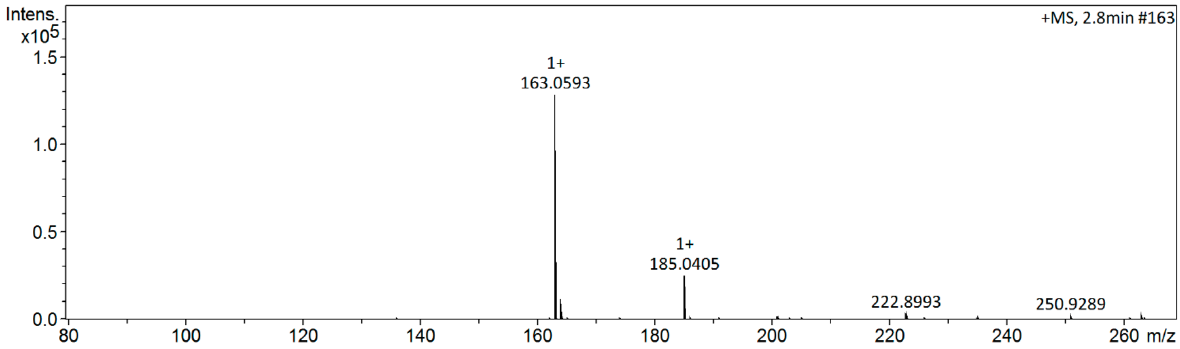

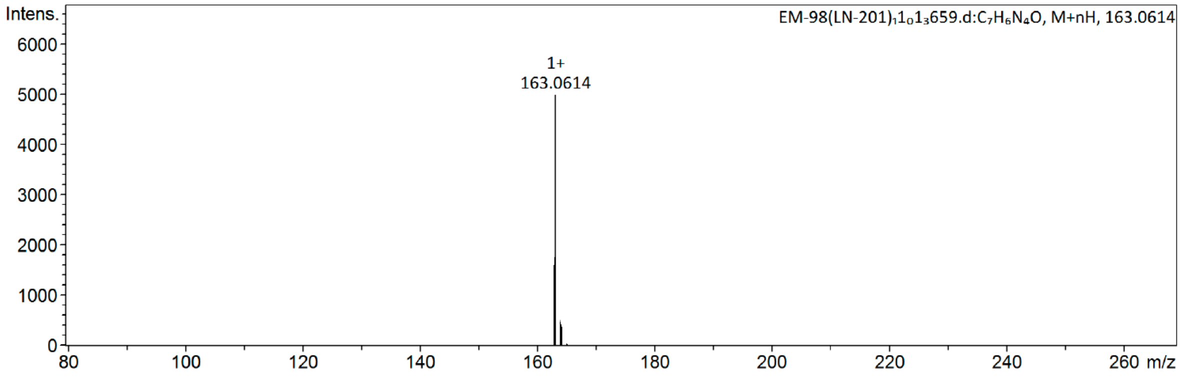

HRMS spectrum of **1b**

Display Report

Analysis Info

Analysis Name	D:\Data\Em-79(FB5)_2_01_3328.d	Acquisition Date	3/27/2020 2:41:34 PM
Method	Ia_2.2_small.m	Operator	BDAL@DE
Sample Name	1b	Instrument	compact 8255754.20088
Comment			

Acquisition Parameter

Source Type	ESI	Ion Polarity	Positive	Set Nebulizer	0.4 Bar
Focus	Active	Set Capillary	4500 V	Set Dry Heater	180 °C
Scan Begin	50 m/z	Set End Plate Offset	-500 V	Set Dry Gas	6.0 l/min
Scan End	3000 m/z	Set Charging Voltage	2000 V	Set Divert Valve	Source
		Set Corona	0 nA	Set APCI Heater	0 °C

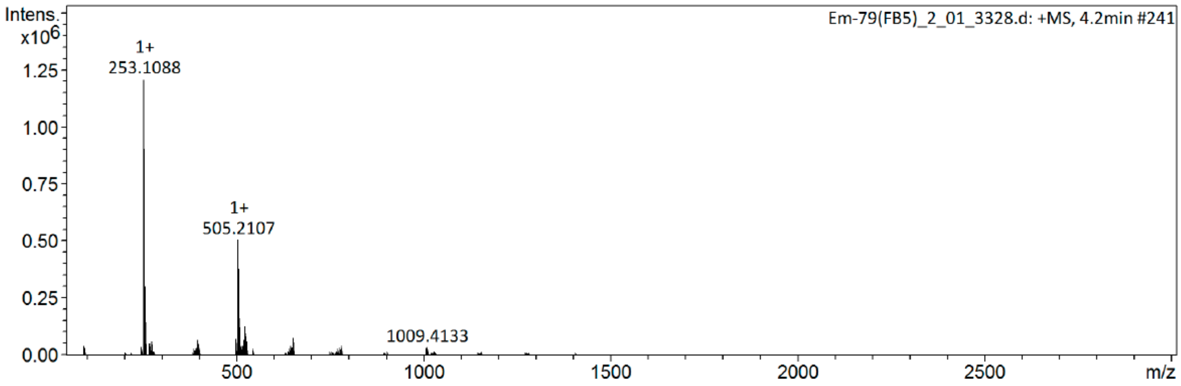

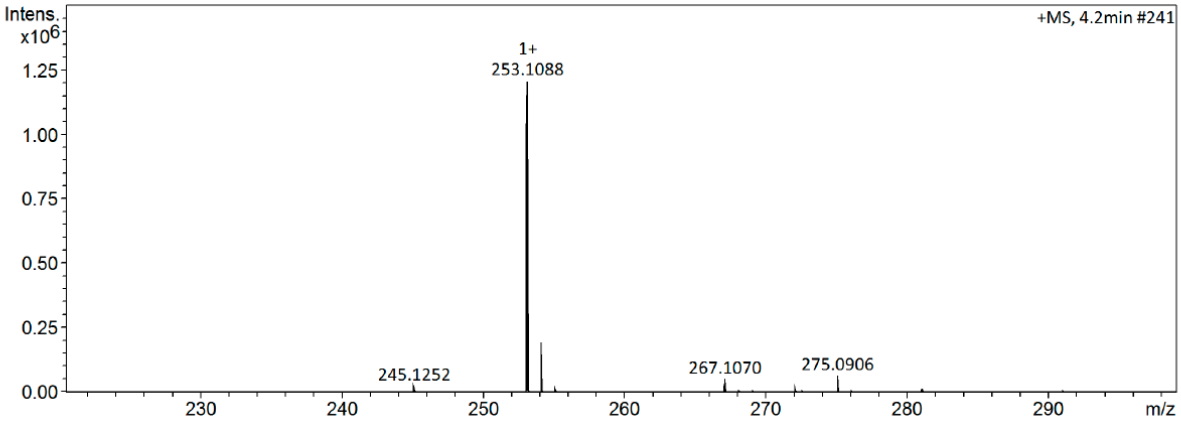

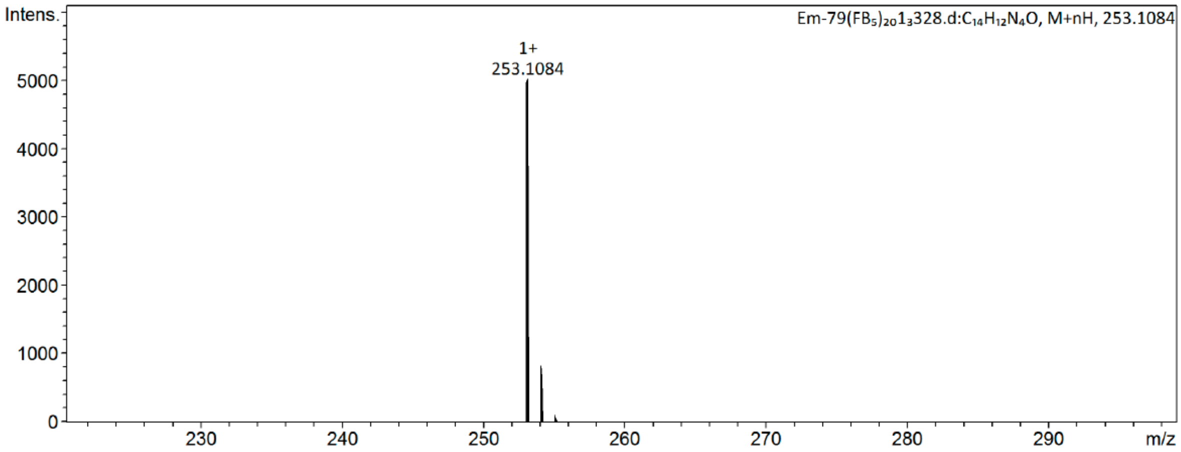

HRMS spectrum of **6**

Display Report

Analysis Info Acquisition Date 07.08.2023 14:51:03

Analysis Name D:\Data\Chizhov\IMB\Matyugina\Aug_07_2023\In-199_&clblow.d
Method tune_low.m Operator BDAL@DE
Sample Name 6 Instrument / Ser# micrOTOF CH3CN : 10248
Comment H2O 50/50 %, dil. 200, calibrant added

Acquisition Parameter

Source Type	ESI	Ion Polarity	Positive	Set Nebulizer	0.4 Bar
Focus	Not active			Set Dry Heater	180 °C
Scan Begin	50 m/z	Set Capillary	4500 V	Set Dry Gas	4.0 l/min
Scan End	2000 m/z	Set End Plate Offset	-500 V	Set Divert Valve	Waste

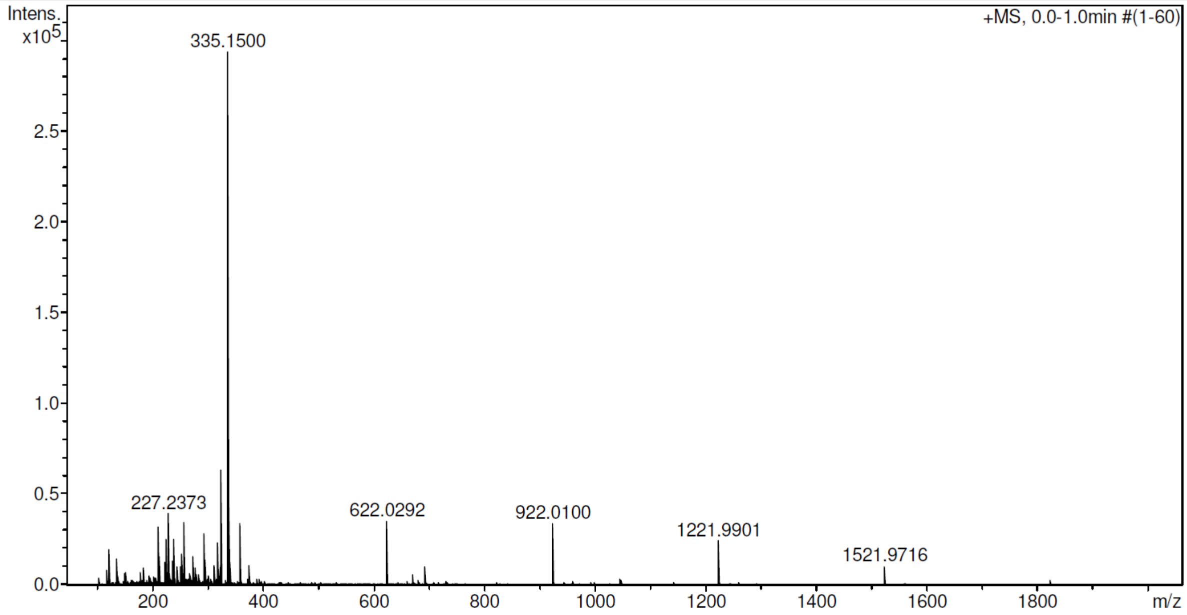

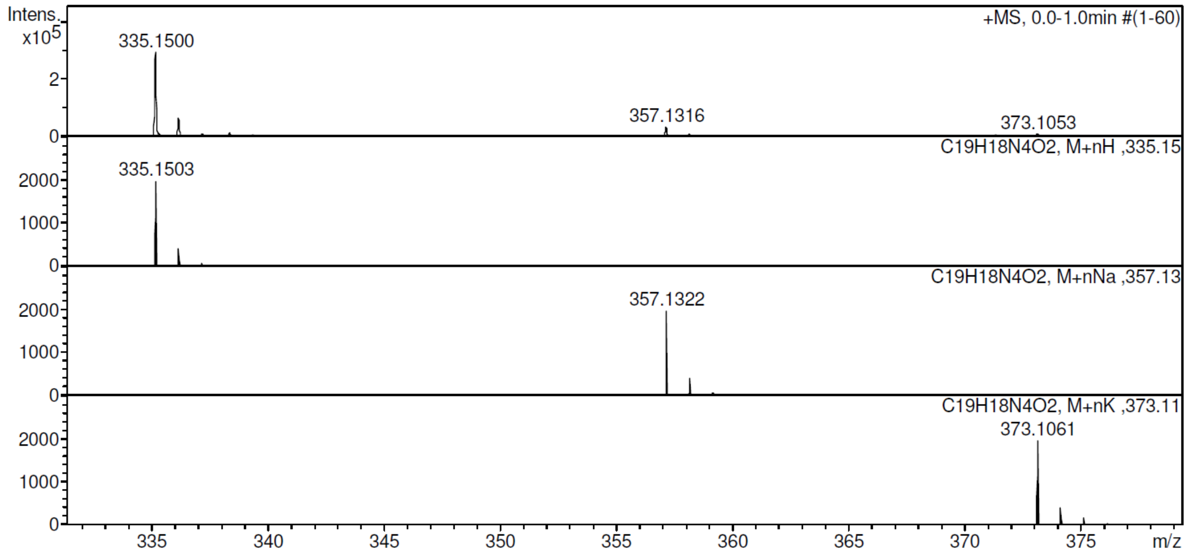

HRMS spectrum of **7**

Display Report

Analysis Info

Analysis Name	D:\Data\EM112_1_01_3888.d	Acquisition Date	9/27/2021 2:52:34 PM
Method	la_2.2_small.m	Operator	BDAL@DE
Sample Name	7	Instrument	compact 8255754.20088
Comment			

Acquisition Parameter

Source Type	ESI	Ion Polarity	Positive	Set Nebulizer	0.4 Bar
Focus	Active	Set Capillary	4500 V	Set Dry Heater	180 °C
Scan Begin	50 m/z	Set End Plate Offset	-500 V	Set Dry Gas	6.0 l/min
Scan End	3000 m/z	Set Charging Voltage	2000 V	Set Divert Valve	Source
		Set Corona	0 nA	Set APCI Heater	0 °C

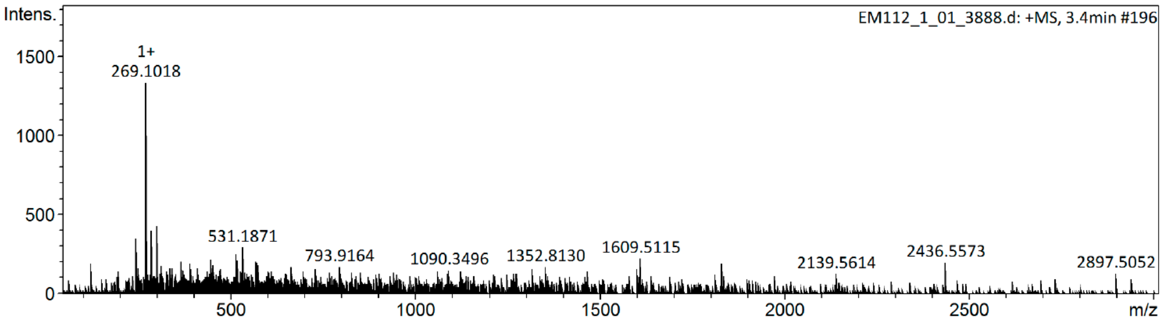

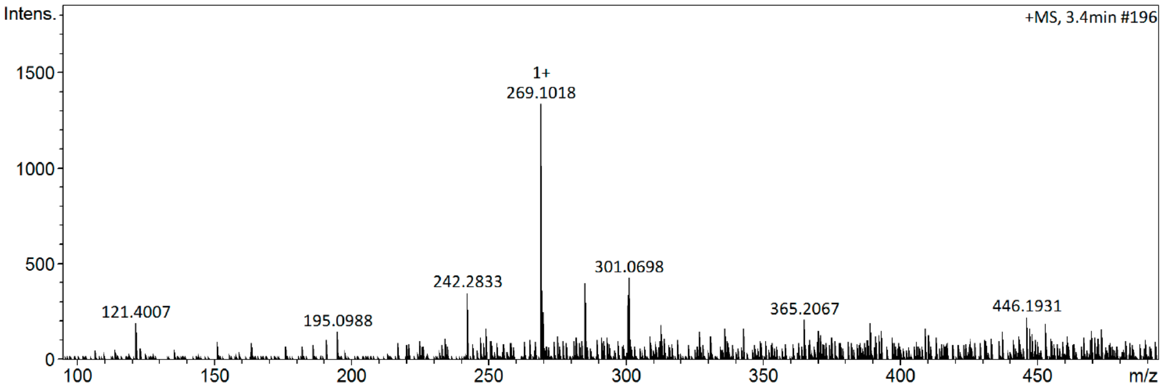

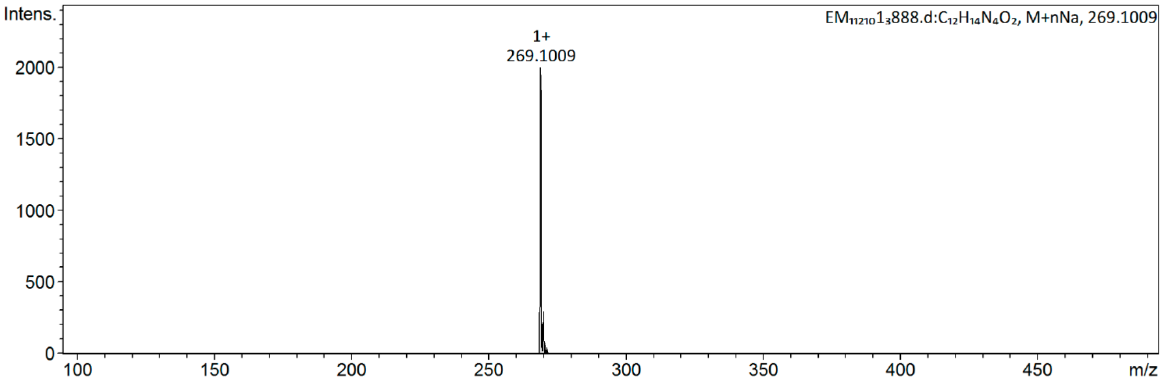

HRMS spectrum of **8**

Display Report

Analysis Info

Analysis Name	D:\Data\Chizhov\IMB\Matyugina\Aug_07_2023\In-220_&clblow.d	Acquisition Date	07.08.2023 14:46:02
Method	tune_low.m	Operator	BDAL@DE
Sample Name	8	Instrument / Ser#	micrOTOF CH3CN : 10248
Comment	H2O 50/50 %, dil. 200, calibrant added		

Acquisition Parameter

Source Type	ESI	Ion Polarity	Positive	Set Nebulizer	0.4 Bar
Focus	Not active			Set Dry Heater	180 °C
Scan Begin	50 m/z	Set Capillary	4500 V	Set Dry Gas	4.0 l/min
Scan End	2000 m/z	Set End Plate Offset	-500 V	Set Divert Valve	Waste

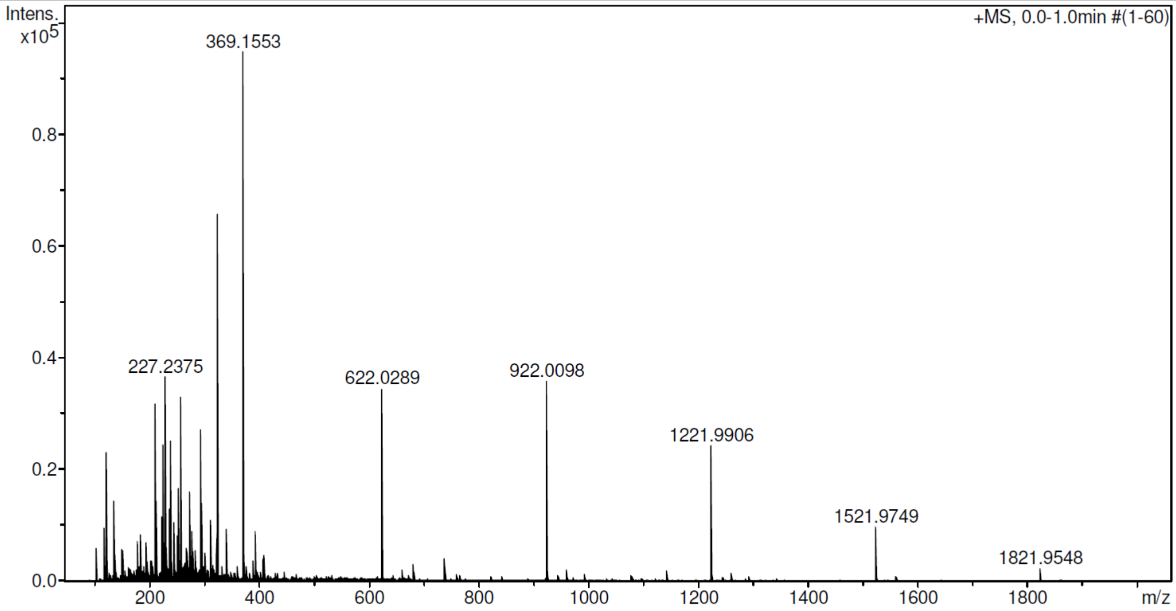

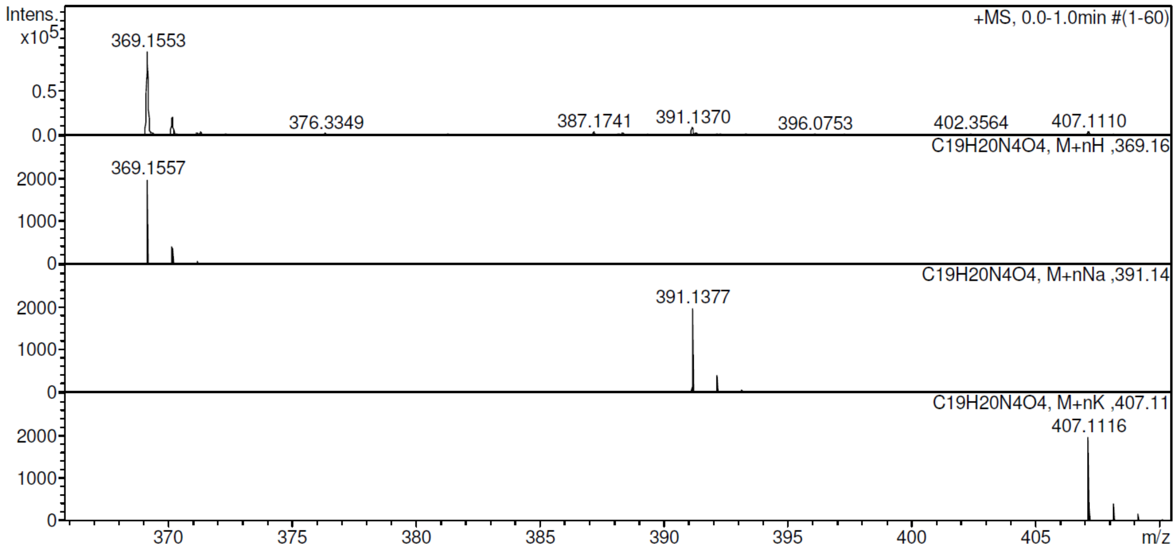

HRMS spectrum of **9**

Display Report

Analysis Info

Analysis Name	D:\Data\Em123-LN261_31_01_4198.d	Acquisition Date	6/23/2023 3:53:39 PM
Method	la_2.2_small2.m	Operator	BDAL@DE
Sample Name	9	Instrument	compact 8255754.20088
Comment			

Acquisition Parameter

Source Type	ESI	Ion Polarity	Positive	Set Nebulizer	0.4 Bar
Focus	Active	Set Capillary	3500 V	Set Dry Heater	180 °C
Scan Begin	50 m/z	Set End Plate Offset	-500 V	Set Dry Gas	4.0 l/min
Scan End	3000 m/z	Set Charging Voltage	2000 V	Set Divert Valve	Source
		Set Corona	0 nA	Set APCI Heater	0 °C

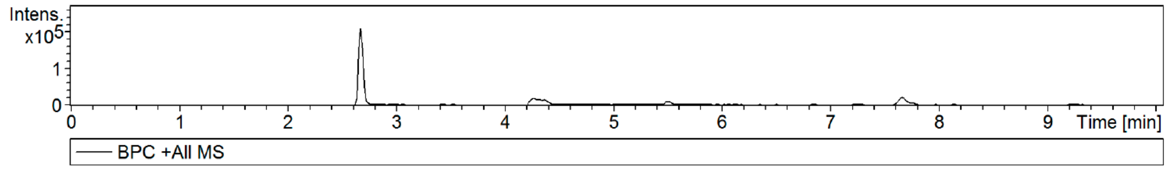

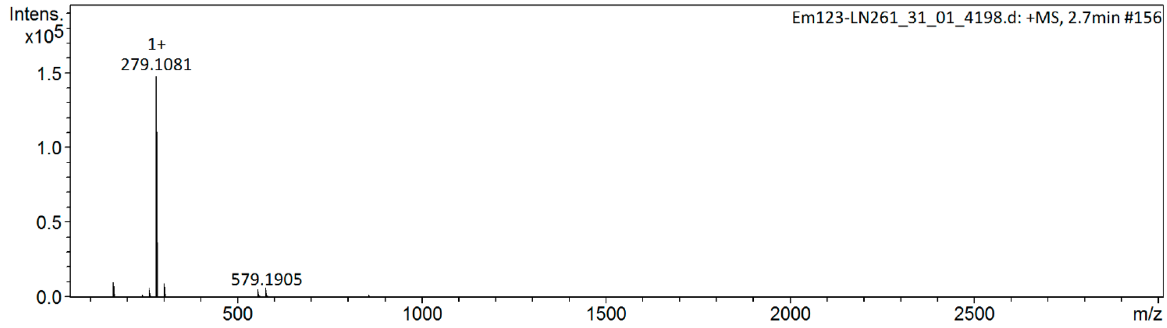

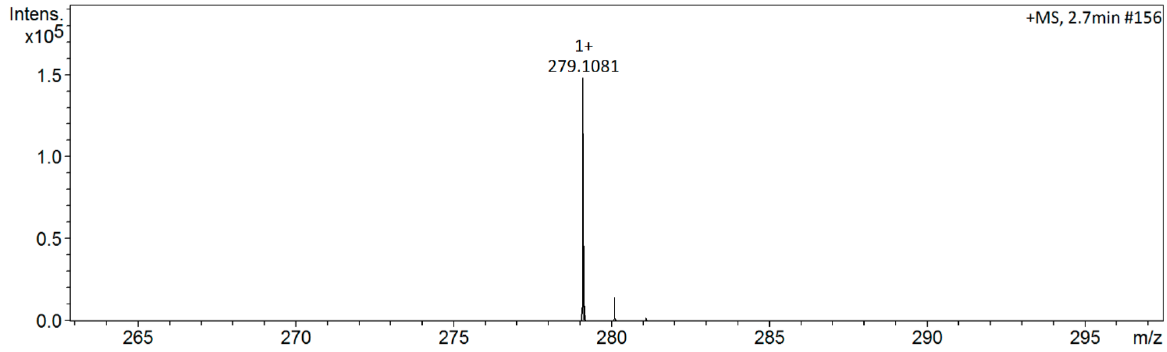

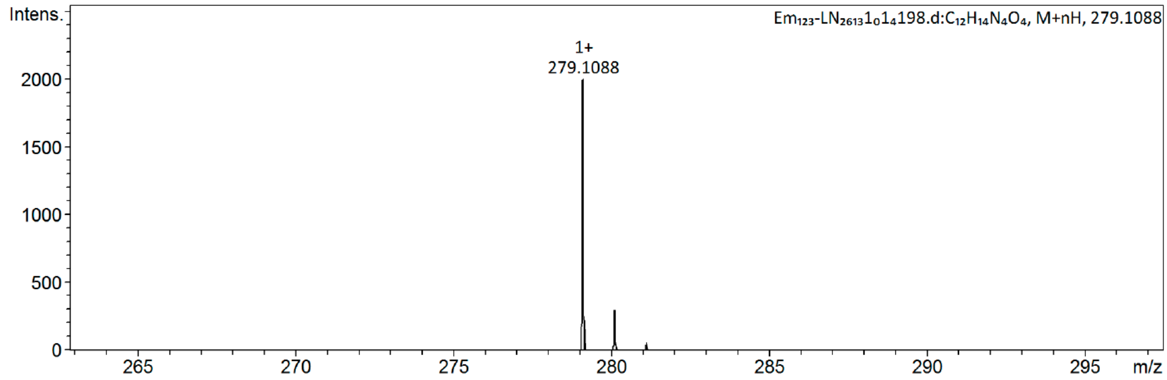

Em123-LN261_31_01_4198.d

Bruker Compass DataAnalysis 4.3 printed: 6/23/2023 4:26:05 PM by: BDAL@DE
